# The *in situ* synthesis of PbS nanocrystals from lead(II) *n*-octylxanthate within a 1,3-diisopropenylbenzene–bisphenol A dimethacrylate sulfur copolymer

**DOI:** 10.1098/rsos.170383

**Published:** 2017-08-16

**Authors:** P. D. McNaughter, J. C. Bear, A. G. Mayes, I. P. Parkin, P. O'Brien

**Affiliations:** 1 School of Chemistry, University of Manchester, Oxford Road, Manchester M13 9PL, UK; 2 School of Materials, University of Manchester, Oxford Road, Manchester M13 9PL, UK; 3 Materials Chemistry Centre, Department of Chemistry, University College London, 20 Gordon Street, London WC1H 0AJ, UK; 4 School of Chemistry, University of East Anglia, Norwich Research Park, Norwich NR4 7TJ, UK

**Keywords:** lead(II) xanthate, sulfur polymer, nanocrystals, inverse vulcanization

## Abstract

The synthesis of lead sulfide nanocrystals within a solution processable sulfur ‘inverse vulcanization’ polymer thin film matrix was achieved from the *in situ* thermal decomposition of lead(II) *n*-octylxanthate, [Pb(S_2_COOct)_2_]. The growth of nanocrystals within polymer thin films from single-source precursors offers a faster route to networks of nanocrystals within polymers when compared with *ex situ* routes. The ‘inverse vulcanization’ sulfur polymer described herein contains a hybrid linker system which demonstrates high solubility in organic solvents, allowing solution processing of the sulfur-based polymer, ideal for the formation of thin films. The process of nanocrystal synthesis within sulfur films was optimized by observing nanocrystal formation by X-ray photoelectron spectroscopy and X-ray diffraction. Examination of the film morphology by scanning electron microscopy showed that beyond a certain precursor concentration the nanocrystals formed were not only within the film but also on the surface suggesting a loading limit within the polymer. We envisage this material could be used as the basis of a new generation of materials where solution processed sulfur polymers act as an alternative to traditional polymers.

## 1. Introduction

**Scheme 1.**
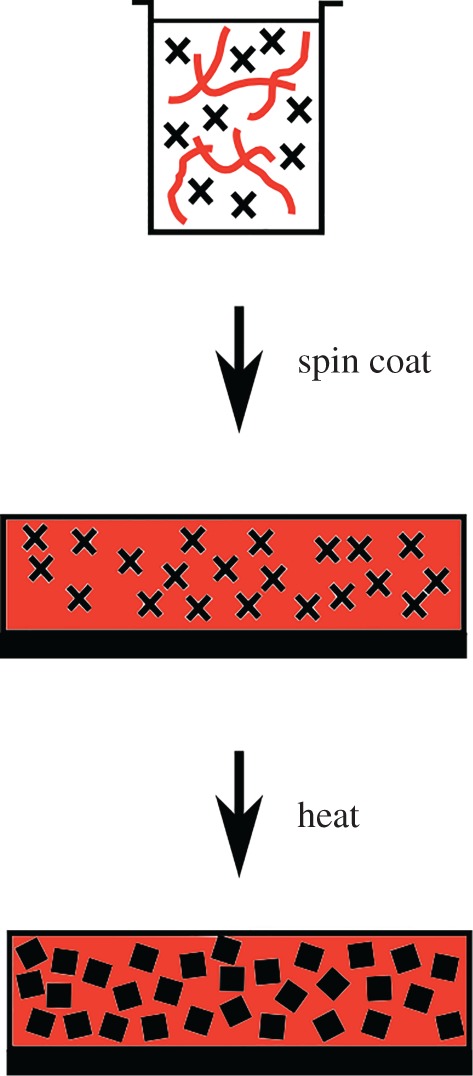
The processing of precursor and polymer to form PbS nanocrystals within the sulfur polymer.

The *in situ* growth of nanomaterials from single-source precursors in polymers offers a potentially shorter route to thin films of bi-continuous phases of inorganic nanocrystals and polymers, by growing the nanocrystals in the polymer rather than synthesizing them *ex situ* followed by incorporation into the polymer. The technique involves the dissolution of a precursor for the inorganic nanocrystals and a polymer in a mutual solvent, before coating a desired substrate [1]. The substrate is then heated causing the decomposition of the precursor to given nanocrystals in the polymer film (scheme 1). This has the advantage over *ex situ* routes as surface ligands, a necessity for colloidal stability, can be avoided thus improving contact between the particles and polymer in the film, a particular advantage when producing bulk heterojunction photovoltaics [1]. The wide range of single-source precursors and polymers available make the growth of nanomaterials directly within polymers an attractive route for the synthesis of composite thin films and has been used for PbS [2], CdS [3–7], Bi_2_S_3_ [8], CuInS_2_ [9,10], Sb_2_S_3_ [11] in photovoltaic and thermoelectric devices, e.g. PbS in semiconducting polymers for photovoltaics. Potential applications for nanocrystal-containing thin films include: solar cells [9,10,12–15], catalysis [16–18], absorptive optical filters [19,20], sensors [21–25] and antimicrobial surfaces [26–29].

Single-source precursors are well established for the production of nanoscale metal chalcogenide thin films and particles due to their ease of synthesis and clean decomposition [30–36]. In the case of lead(II) sulfide precursors [32], examples of such species include: xanthates [37–40], dichalcogocarbamates [41–43], acylchalcogoureas [42,44], dichalcogenophosphinates [45], dichalcogenophosphates [46] and dichalcogenidoimidophosphinates [47,48]. In particular, metal xanthates are ideal for decomposition within polymers and melts due to their volatile by-products produced from the Chugeav elimination, i.e. H_2_S, SCO and an alkene. At the breakdown temperatures used the products are not found within the films produced.

The use of sulfur as a feedstock for polymeric materials has arisen due to the abundance of elemental sulfur, with approximately 60 million tons produced annually [49–51]. Sulfur polymers are known as ‘inverse vulcanization’ materials due to the reaction of elemental sulfur at elevated temperatures with divinylic species [52–54], forming sulfur chains cross-linked with organic linkers, in contrast with traditional vulcanization used to strengthen rubber where poly(isoprene) chains are cross-linked with di-sulfide bridges. These polymeric materials have potential as battery materials [55,56], infrared lenses [57,58] and as effective filters for heavy metal ions [59–61] due to their ease of manufacture, chemical inertness and optical translucence. These materials are worth investigating due to the large quantity of sulfur waste created from petroleum refining, thus alleviating pressure on traditional polymer feedstocks, namely crude oil.

We present the synthesis of PbS nanocrystals in a solution processable sulfur polymer from the *in situ* decomposition of lead(II) *n*-octylxanthate, [Pb(S_2_COOct)_2_]. This new material has the potential to replace conventional polymer–nanocrystal materials by taking advantage of elemental sulfur as an alternative feedstock. The nanocrystal–polymer thin films have potential as absorptive optical filters, or colour gels, where the traditionally used polycarbonate or polyester has been replaced with a solution processable sulfur polymer. By variation of the ratio of polymer to precursor prior to spin coating, decomposition temperature and polymer composition, the influence on film morphology has been explored. The suitability of the sulfur polymer for thin film formation was optimized through the addition of bisphenol A dimethacrylate to the 1,3-diisopropenylbenzene linker, which raised the melting and decomposition temperatures as well as the hardness of the polymer, a key advantage for the formation of robust polymer–nanocrystal thin films [62].

## 2. Material and methods

### 2.1. Reagents

Sulfur (98%), sodium hydride (dry, 95%), *n*-octanol (anhydrous, greater than or equal to 99%), carbon disulfide (low benzene, greater than or equal to 99.9%), lead acetate trihydrate (greater than or equal to 99.9%) and bisphenol A dimethacrylate (greater than 98%) were purchased from Sigma-Aldrich Ltd. 1,3-Diisopropenylbenzene (greater than 97.0%) was purchased from TCI Chemicals and potassium hydroxide (greater than 85%) was purchased from Fisher Scientific Ltd.

Solvents were purchased from Sigma-Aldrich Ltd and used as received. Deionized (DI) water with a resistivity of not less than 18.2 MΩ cm^−1^ (Millipore, UK) was used for aqueous solutions.

### 2.2. Methods

#### 2.2.1. Synthesis of lead(II) *n*-octylxanthate, [Pb(S_2_COOct)_2_]

Sodium hydride (1.2 g, 50 mmol) and *n*-octanol (7.89 ml, 50 mmol) were mixed with 50 ml of diethyl ether at 0°C. Carbon disulfide (3.0 ml, 50 mmol) was added dropwise under vigorous stirring giving a yellow solution. After approximately 1 h equilibration at room temperature, lead acetate trihydrate (9.48 g, 25 mmol) dissolved in 50 ml of DI water was added dropwise, forming a cream-coloured precipitate. The precipitate was collected by filtration, washed with DI water and dried *in vacuo*. Elemental analysis calculated for [Pb(S_2_COOct)_2_]: C, 34.99; H, 5.55; S, 20.76. Found: C, 35.01; H, 5.61; S, 20.73. ^1^H NMR (400 MHz, CDCl_3_) *δ* = 4.63 (t, *J* = 6.7 Hz, 4H; CH_3_), 1.94−1.80 (m, 4H; CH_2_), 1.47−1.36 (m, 4H; CH_2_), 1.36−1.20 (m, 16H), 0.88 (t, *J *= 6.9 Hz, 6H; CH_3_). M.p. = 77−78°C.

#### 2.2.2. Synthesis of poly(sulfur-*co*-1,3-diisopropenylbenzene), 50 wt% ratio of linker (**1**)

Poly(sulfur-*co*-1,3-diisopropenylbenzene) was prepared according to Chung *et al.* [62]. Briefly, elemental sulfur (S_8_, 2 g, 7.81 mmol) was added to a glass vial and heated to 185°C in a graphite bath under vigorous stirring. Once 185°C was reached, 1,3-diisopropenylbenzene (2.16 ml, 12.6 mmol) was injected, the whole mixture agitated with a glass rod and stirred for 4–5 min. The temperature was then raised to 200°C and the polymer was allowed to cure at that temperature for 30 min.

#### 2.2.3. Synthesis of poly(sulfur-*co*-bisphenol A dimethacrylate) (**2**)

Poly(sulfur-*co*-bisphenol A dimethacrylate) was synthesized using the procedure for (**1**), except that bisphenol A dimethacrylate (4.59 g, 12.6 mmol) was used instead of 1,3-diisopropenylbenzene, and vigorous mechanical stirring was required to ensure full mixing of the monomer and the molten sulfur.

#### 2.2.4. Synthesis of poly(sulfur-*co*-bisphenol A dimethacrylate-*co*-1,3-diisopropenylbenzene) (**3**) and (**4**)

Poly(sulfur-*co*-bisphenol A dimethacrylate-*co*-1,3-diisopropenylbenzene) was synthesized using the procedure outlined for poly(sulfur-*co*-bisphenol A dimethacrylate), except that differing ratios of the 1,3-diisopropenylbenzene and bisphenol A dimethacrylate linkers were used. For (**3**), S_8_ (4 g, 15.6 mmol), bisphenol A dimethacrylate (2.25 g, 6.17 mmol) and diisopropenylbenzene (3 ml, 17.5 mmol) and for (**4**), S_8_ (4 g, 15.6 mmol), bisphenol A dimethacrylate (6.38 g, 17.5 mmol) and diisopropenylbenzene (1.06 ml, 6.17 mmol) were used.

#### 2.2.5. Synthesis of poly(sulfur-*co*-linker)-lead sulfide thin films

Poly(sulfur-*co*-linker)-lead sulfide thin films were synthesized using a protocol outlined by Lewis *et al.* [2]. Briefly, poly(sulfur-*co*-bisphenol A dimethacrylate-*co*-1,3-diisopropenylbenzene) (85 mg) (**4**) was dissolved in chloroform (2.5 ml) by heating and swirling at 50°C for an hour. [Pb(S_2_COOct)_2_] (60 mg, 0.1 mmol) was then added, resulting in a dark brown solution. Glass slides (20 × 15 × 1 mm) were cleaned by sonication in 2-propanol, and dried in air. A total of 100 µl of the lead(II) *n*-octylxanthate-polymer solution was coated onto the glass slide by spin coating at 1250 r.p.m. for 20 s followed by 4000 r.p.m. for 10 s. The films were loaded into a quartz tube for heating and placed under N_2_ using standard Schlenk techniques. The tube was inserted into the furnace preheated at 150°C and held at the chosen temperature for the specified time. Once finished, the tube was removed from the furnace and allowed to cool in air taking approximately 3 min.

### 2.3. Instrumentation

High-resolution transmission electron microscopy (HRTEM) measurements were conducted using an FEI Tecnai G20 TEM with a LaB_6_ source operating at an acceleration voltage of 200 kV. EDS spectra were taken with an Oxford XMax 80 TLE. TEM samples were prepared dissolving the films in 1,2-dichlorobenzene followed by drop casting. UV/visible absorption spectra were recorded using a Shimadzu UV-1800 instrument over a range of 350–1000 nm. X-ray photoelectron spectroscopy (XPS) was undertaken using a Thermo Scientific K-alpha spectrometer with monochromated Al Kα radiation, a dual beam charge compensation system and constant pass energy of 50 eV (spot size 400 µm). Survey scans were collected in the range of 0–1200 eV. High-resolution peaks were used for the principal peaks of S (2p), O (1 s) and C (1 s) and fitted with CASA XPS software. Elemental analysis, thermogravimetric analysis (TGA)/differential scanning calorimetry (DSC) and temperature cycling DSC were performed by the University of Manchester Microanalytical Laboratory on a Mettler Toledo TGA/DSC 1 Stare System (between 30°C and 1000°C) and a Perkin Elmer—Jade DSC (between 0°C and 200°C), respectively. Grazing incidence X-ray diffraction (XRD) patterns were acquired using a Bruker D8 Advance diffractometer, using a Cu Kα (*λ* = 1.5418 Å) source with a Göbel mirror optic and Soller slits on the detector side. Scans were acquired at a grazing incidence of 3° over a 2*θ* range of 10−80° with 0.02° steps and 3 s per step. Scanning electron microscopy (SEM) images were acquired using an FEI Quanta 650 SEM using an accelerating voltage of 20 kV.

## 3. Results and discussion

### 3.1. Solution processable sulfur polymer synthesis and characterization

**Scheme 2.**
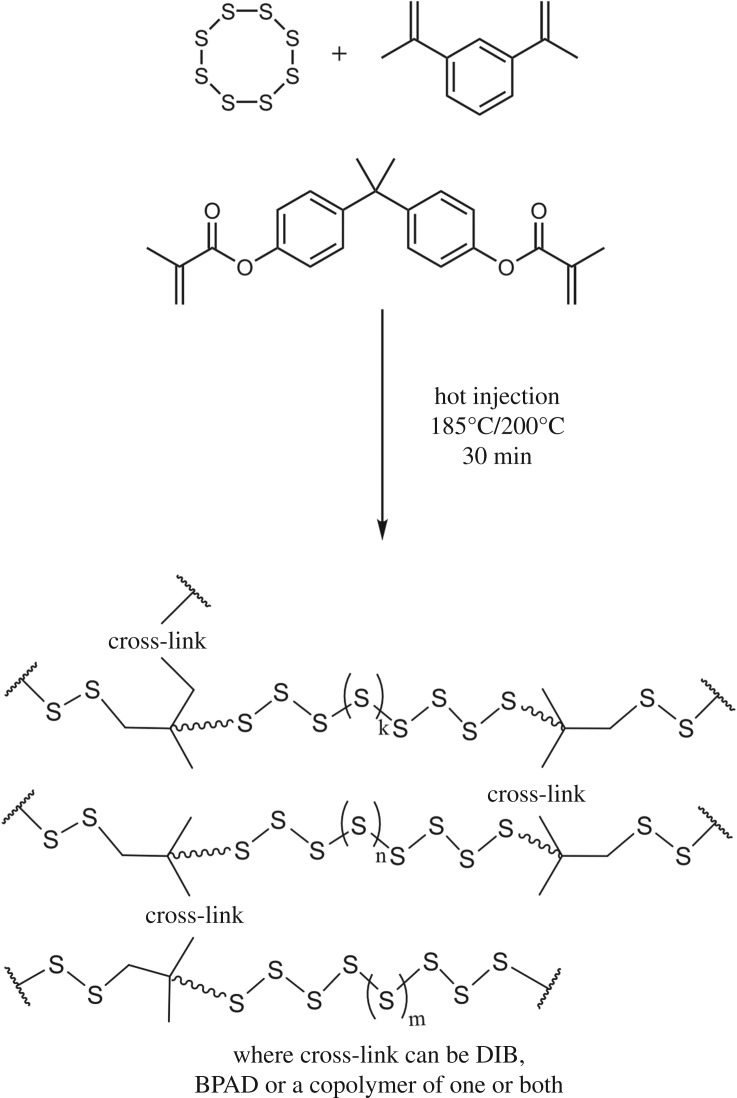
The ‘inverse vulcanization’ polymerization used to produce the sulfur-based polymer cross-linked with 1,3-diisopropenylbenzene and bisphenol A dimethacrylate.

Bisphenol A dimethacrylate was reacted with sulfur in conjunction with 1,3-diisopropenylbenzene in an ‘inverse vulcanization’ process to create a sulfur-based polymer that was more robust with a higher melting point and decomposition temperature than pure poly(sulfur-*co*-1,3-diisopropenylbenzene), shown in scheme 2. This combination of cross-linkers results in the increased solubility in organic solvents due to the formation of a suspected hyperbranched structure allowing solution processing [63]. Derivatives of bisphenol A are a common component of epoxy resins, acting as the cross-linking polymer backbone to hardeners such as aliphatic amines in the epoxy blend [64]. It was found that molten sulfur and bisphenol A dimethacrylate exhibit poor miscibility, with constant, strong mechanical agitation required to form the polymer. The miscibility improved upon addition of 1,3-diisopropenylbenzene to the sulfur/bisphenol A dimethacylate mixture.

On heating, yellow sulfur S_8_ rings melt between 120°C and 124°C [65,66] before the critical temperature of approximately 159°C, the *λ*-transition, at which the specific heat capacity, viscosity, density and thermal expansion all change dramatically. The changes are due to the ring opening of S_8_, forming long-chain oligomeric short and long [–S–S–]_x_ chains with radical end groups [62]. This is accompanied by a darkening in colour to red potentially due to organic impurities within the sulfur [62,65]. The radical end-groups react with divinylic species of the bisphenol A dimethacrylate and 1,3-diisopropenylbenzene forming the ‘inverse vulcanization’ sulfur polymer. NMR spectra of the monomers and the sulfur copolymers showed incorporation of the aromatic regions from the cross-linkers and the disappearance of the alkene region indicating polymerization had occurred (see electronic supplementary material, figure S1). The polymerization was also confirmed by Fourier transform IR spectroscopy (see electronic supplementary material, figures S2 and S3). The IR spectrum of 1,3-diisopropenylbenzene shows three peaks corresponding to C=C absorptions at 1628.8, 1594.8 and 1573.5 cm^−1^ (see electronic supplementary material, figure S3*a*). The absorption at 1628.8 cm^−1^ corresponds to the unsaturated alkene with lower wavenumber peaks coming from the benzene ring. Upon polymerization with elemental sulfur the alkene peak at 1628.8 cm^−1^ disappears and the benzene C = C absorptions shift to 1601.5 cm^−1^ and 1584.2 cm^−1^ caused by the reduction in the size of the aromatic system following polymerization (see electronic supplementary material, figure S3*f*). The IR spectrum of unreacted bisphenol A dimethacrylate possesses a carbonyl stretch at 1726.5 cm^−1^ (electronic supplementary material, figure S3*b*) which upon reaction of the adjacent alkene causes the carbonyl stretch to shift to 1746.7 cm^−1^ as expected with the size reduction of the aromatic system following polymerization (see electronic supplementary material, figure S3*e*). The C = C of the alkene at 1634.4 cm^−1^ in bisphenol A dimethacrylate disappears and the C= C of the benzene shifts from 1600.4 to 1648.8 cm^−1^ when polymerized. The S-polymers using both cross-linkers, (**3**) and (**4**), display the shifted aromatic C–C stretches for both of the cross-linkers and also the shift in the BPAD carbonyl signal as observed in the polymers using only a single cross-linker (see electronic supplementary material, figure S3*c*,*d*).

**Figure 1.**
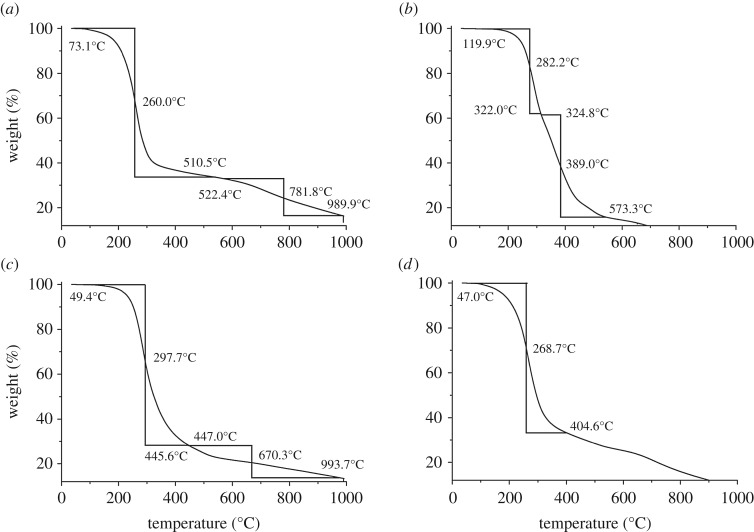
TGA profiles of: (*a*) pure poly(sulfur-*co*-1,3-diisopropenylbenzene) (**1**), (*b*) pure poly(sulfur-*co*-bisphenol A dimethacrylate) (**2**), (*c*) poly(sulfur-*co*-bisphenol A dimethacrylate-*co*-1,3-diisopropenylbenzene), higher ratio of 1,3-diisopropenylbenzene (**3**) and (*d*) poly(sulfur-*co*-bisphenol A dimethacrylate-*co*-1,3-diisopropenylbenzene), higher ratio of bisphenol A dimethacrylate (**4**).

TGA and DSC were used to examine the thermal properties of the copolymers and showed that the addition of the bisphenol A increases the onset of decomposition for the polymer (figure 1). For example, the melting point range (from capillary melting point apparatus) of pure poly(sulfur-*co*-1,3-diisopropenylbenzene) (**1**) at 50 wt% organic content is 15°C–35°C compared to that of 190°C–245°C (starts to decompose) for pure poly(sulfur-*co*-bisphenol A dimethacrylate) (**2**). Composite polymers (**3**) and (**4**) were shown to have intermediate melting temperatures (180°C–200°C for (**3**) and 205°C–225°C for (**4**)), commensurate with the molar ratios of 1,3-diisopropenylbenzene to bisphenol A dimethacrylate.

Transition temperatures were determined from the DSC traces in electronic supplementary material, figure S4. There is a large decomposition step present in all of the polymer traces: onset 228.5°C for polymer (**1**), 250.7°C for polymer (**2**), 263.5°C for polymer (**3**) and 247.4°C for polymer (**4**) commensurate with elemental sulfur leaching out of the structure and carbonization [61] as well as direct evidence that the presence of bisphenol A dimethacrylate contributes to the thermal stability of the polymer. Owing to the presence of this decomposition at temperatures less than 200°C, DSC thermal cycles were undertaken between 0°C and 200°C at a heating rate of 10°C min^−1^ before cooling to 0°C at 10°C min^−1^ before undertaking a second heating cycle. Polymer (**1**) showed a small feature at 32.2°C which was assigned as the *T*_g_ of the polymer, and is comparable with the *T*_g_ observed for the 50 wt% 1,3-diisopropenylbenzene sulfur polymer by Pyun *et al.* [62] of 28.4°C (see electronic supplementary material, figures S5 and S6). The DSC curves for polymers (**2**), (**3**) and (**4**) showed no *T*_g_ peaks on the heating or cooling curves, and only small melting transitions observed on the heating curves. There were no corresponding peaks on the cooling curves for polymers (**3**) and (**4**), indicating the properties of the material do not change with temperature until the onset of decomposition (see electronic supplementary material, figures S7, S8 and S9).

Powder XRD of polymers (**1**)–(**4**) showed no reflections indicating that the polymers are amorphous and that the polymerization has gone to completion as no residual S_6_ or S_8_ is present (see electronic supplementary material, figure S10).

The solubility of a polymer is of vital importance for applications that require solution processing. Bear *et al.* [50] reported that higher sulfur content poly(sulfur-*co*-1,3-diisopropenylbenzene) was insoluble in all organic solvents except for very limited solubility in 1,2-dichlorobenzene. When linker content is near 50 wt%, solubility in chlorinated solvents increases, making chloroform an ideal solvent for dissolution of the polymer and allows solution processing with [Pb(S_2_COOct)_2_]. Work by Pyun *et al.* indicated that the reactor in which the ‘inverse vulcanization’ polymer was synthesized played an important role in the solubility of the 1,3-diisopropenylbenzne-sulfur polymers [57]. Other groups have published examples of ‘inverse vulcanization’ polymers with solubility in a wider range of organic solvents using different linkers including: divinylbenzene [67], allyl functionalized benzoxazine [68], S-farnesol [69] and S-limonene [60]. In this work, polymers (**1**) and (**3**) exhibited solubility in a wide range of organic solvents including: *n*-hexane, 1,2-dichlorobenzene, chloroform, toluene and dichloromethane. Polymers (**2**) and (**4**) dissolved partially, leaving a transparent, insoluble loose residue in an otherwise clear orange solution. These polymers had a high weight fraction of bisphenol A dimethacrylate, and potentially difficulties in obtaining a homogeneous reaction mixture may have contributed to poor incorporation of bisphenol A dimethacrylate causing the partial solubility.

Polymer (**1**), however, has a *T*_g_ close to room temperature [62] and is very tacky, which limits the use for formation of robust thin films. Upon heating (**1**) did not withstand the elevated temperatures required for the rapid decomposition of [Pb(S_2_COOct)_2_]. The only PbS nanocrystal–polymer film created using (**1**) was annealed at 70°C for 72 h, yielding a film with irregular substrate coverage (see electronic supplementary material, figure S11). Therefore, polymer (**3**) was used for temperature and concentration studies in PbS nanocrystal–polymer film formation.

### 3.2. Polymer thin film synthesis and characterization

The influence of breakdown temperature was investigated by fixing the S-polymer:[Pb(S_2_COOct)_2_] mass ratio at 6:1 (90 mg or 0.15 mmol of xanthate). Four identical films and a polymer control of (**3**) were heated at 50°C, 100°C and 150°C for 15 min. The effect of varying the [Pb(S_2_COOct)_2_] concentration was also investigated, with 0.05, 0.1, 0.15 and 0.2 mmol of precursor heated at 150°C for 15 min.

### 3.3. Variable temperature study

**Figure 2.**
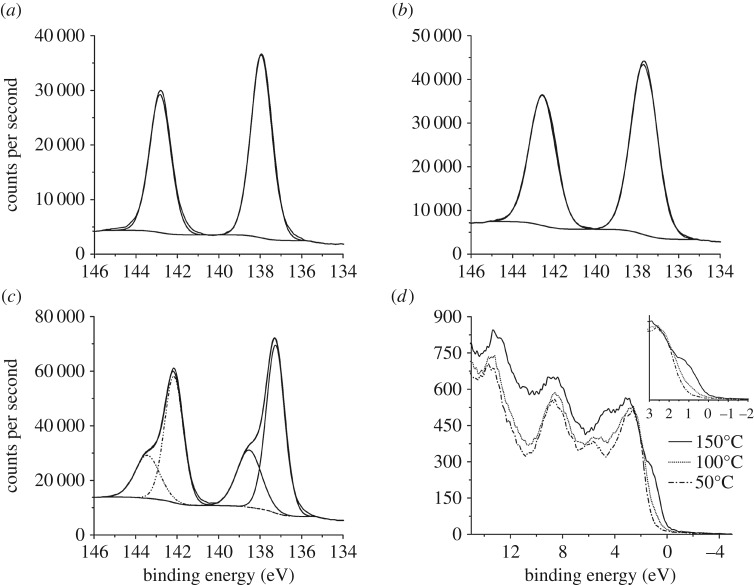
Pb 4f high-resolution XPS spectra are shown for films produced when decomposing [Pb(S_2_COOct)_2_] at: (*a*) 50°C, (*b*) 100°C and (*c*) 150°C. XPS valence spectra (*d*) showing the variation in the position of the Fermi level with heating temperature.

A series of decomposition temperatures (namely 25°C (room temperature), 50°C, 100°C and 150°C) were investigated to ensure the complete breakdown of precursor within a fixed time, i.e. 15 min, and the progress of the reaction was followed using XPS and XRD. XPS for all heated films demonstrated characteristic peaks for lead(II) sulfide, with added sulfur environments attributed to the sulfur polymer, *vide infra*. The valence spectra of the variable temperature samples showed a change in the Fermi level from the precursor-in-polymer control to the 150°C sample, which is characteristic of lead sulfide, figure 2*d* [70].

The Pb 4f XPS spectrum, figure 2*c*, was typical of the composite films heated at 150°C, regardless of the ratio of [Pb(S_2_COOct)_2_] to polymer. The envelope exhibited two environments, with the main region at 137.3 eV (Pb4f_7/2_) assigned as PbS (galena) [71–75] and the second region (138.6 eV) as a lead sulfate, PbSO_x_ [76]. With increasing temperature, the binding energy of the Pb4f_7/2_ region decreases: 138.0 eV (50°C), 137.7 eV (100°C) and 137.3 eV (150°C). This is due to the decomposition of a greater proportion of [Pb(S_2_COOct)_2_] at higher temperatures and the resulting change in the overall Pb chemical environment.

The S2p region of the XPS spectra with variable temperature allows monitoring of the decomposition of [Pb(S_2_COOct)_2_] at the different heating temperatures (see electronic supplementary material, figure S12). Electronic supplementary material, figure S12 shows S2p scans from sulfur in the native polymer (S2p_3/2_, 167.6 eV) and scans from the different temperatures which show the emergence of another feature from the formation of PbS (160.2 eV). A fitted example of an S2p region, electronic supplementary material, figure S13, after heating at 150°C, contains fitted regions for PbS (160.8 eV), elemental sulfur (S_8_, 163.5 eV) and SO_x_ (167.6 eV) (quoted values are position of S2p_3/2_) [71,72,77]. The regions attributed to PbS are not observed below 100°C.

**Figure 3.**
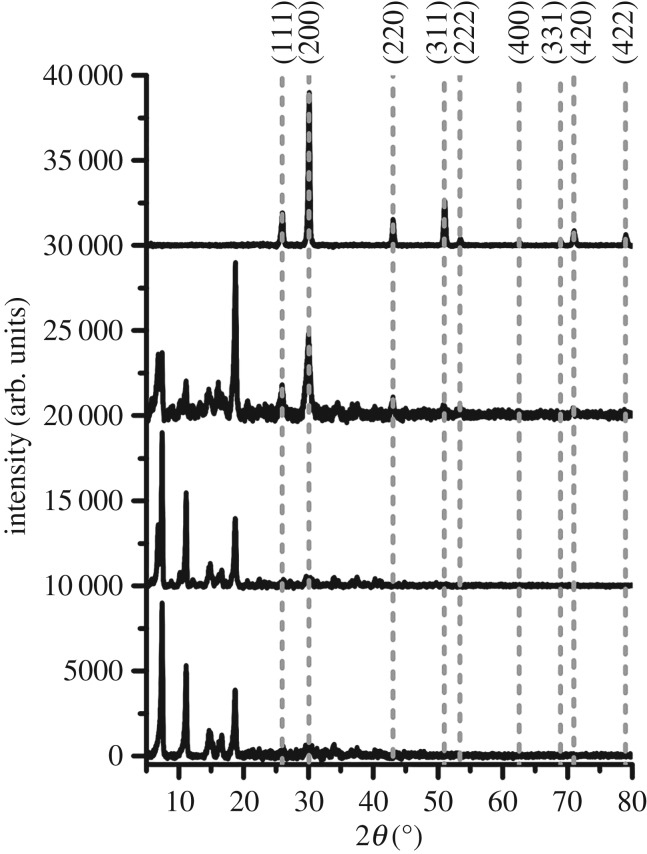
XRD patterns from the films of [Pb(S_2_COOct)_2_] decomposed within the S-polymer (from bottom to top): unreacted control, 50°C, 100°C and 150°C. Dashed lines represent the XRD pattern of PbS (galena), ICSD: 00-005-0592.

The formation of PbS was also followed using XRD (figure 3). The decomposition temperature had a marked effect on the amount of PbS produced in the 15 min heating time, with 150°C yielding phase-pure lead sulfide. At 50°C, only reflections from unreacted [Pb(S_2_COOct)_2_] in S-polymer were found; 100°C showed intermediate decomposition and 150°C showed complete decomposition with no reflections from the precursor present.

### 3.4. Variable concentration study

Different concentrations of [Pb(S_2_COOct)_2_] were investigated when decomposed in (**4**) to investigate the effect on film and nanocrystal morphology. From the variable temperature study, a decomposition temperature of 150°C and a reaction time of 15 min were used to achieve complete precursor decomposition. In all cases, XRD patterns yielded reflections for PbS (see electronic supplementary material, figure S14), and identical XPS spectra to those obtained for the variable temperature study at 150°C. EDS analysis was also consistent, showing the presence of PbS (see electronic supplementary material, figure S15).

**Figure 4.**
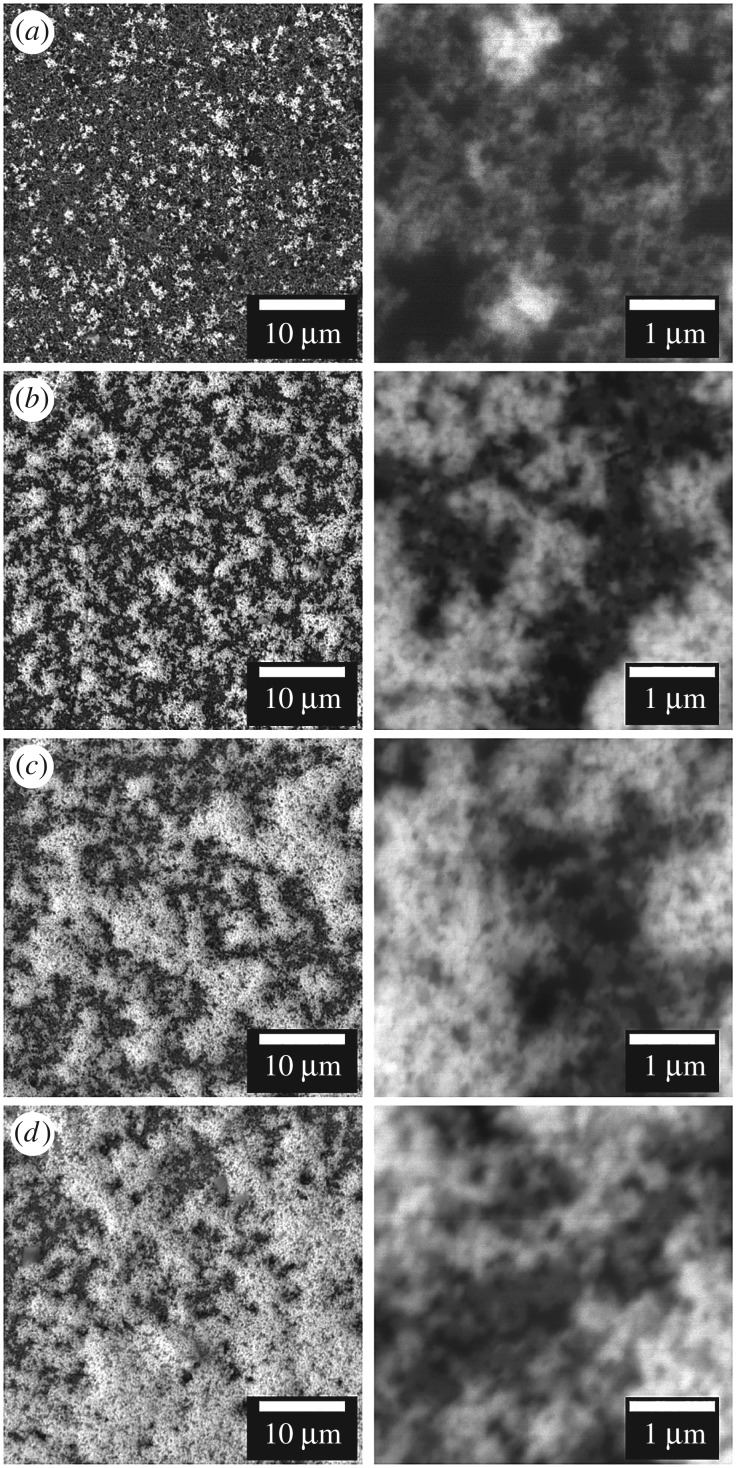
BSE-SEM images at two magnifications of the PbS nanocrystal networks formed within the S-polymer when using (*a*) 0.05 mmol, (*b*) 0.1 mmol, (*c*) 0.15 mmol and (*d*) 0.2 mmol of [Pb(S_2_COOct)_2_].

Backscatter electron (BSE) SEM was used to examine the morphology of the networks of PbS nanocrystals formed within the sulfur polymer films (figure 4). Use of the BSE allows for greater Z contrast and a deeper sampling region, ideal for the examination of Pb-containing nanocrystals within a polymer film. Figure 4*a*–*d* shows the influence on the resulting film of increasing the concentration of the lead(II) octylxanthate. The proportion of PbS nanocrystal rich domains, shown as white regions, increases from figure 4*a* to 4*d* as the amount of [Pb(S_2_COOct)_2_] increases from 0.05 to 0.2 mmol (30–120 mg). The increased proportion of PbS nanocrystal rich sites increases in parallel with the observed increase in film reflectivity as seen in the photographs (see electronic supplementary material, figure S11). The SEM images show that the nanocrystals are incorporated within the S-polymer in the same manner as when grown in polystyrene according to our previous report [2].

**Figure 5.**
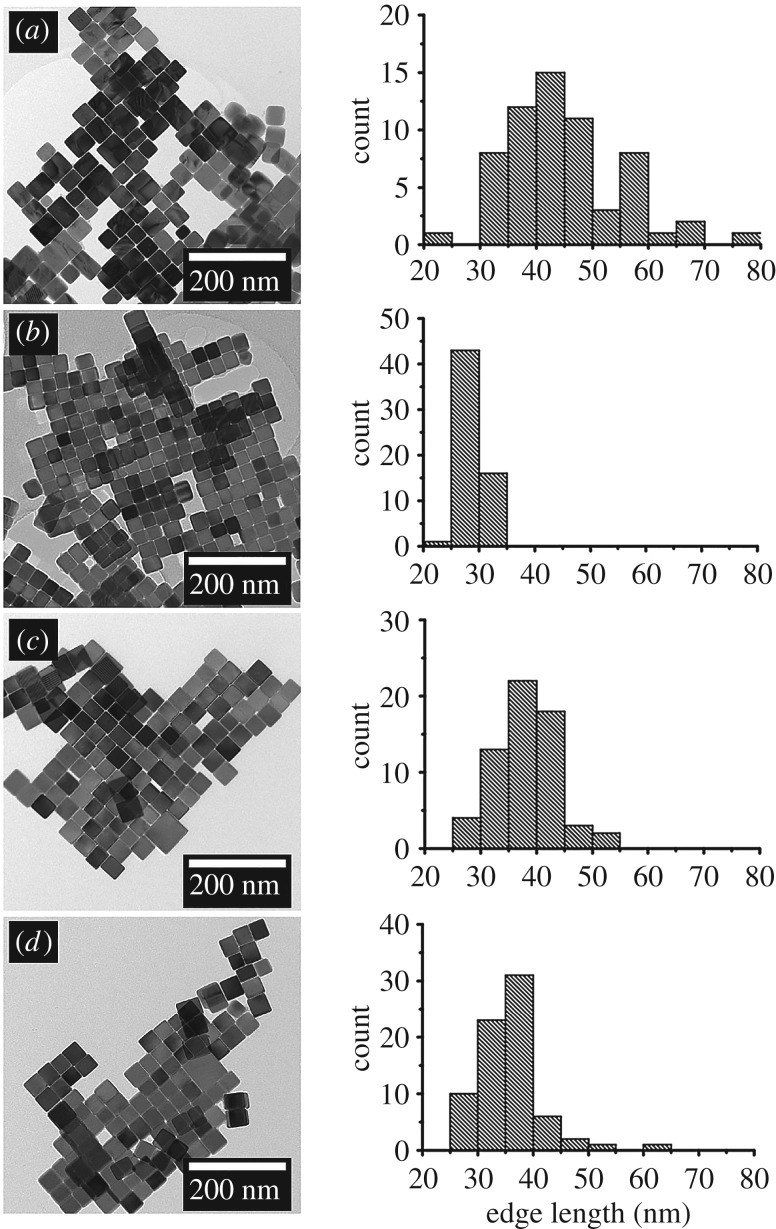
TEM images and corresponding size analysis of the extracted PbS nanocrystals formed within the S-polymer when using (*a*) 0.05 mmol, (*b*) 0.1 mmol, (*c*) 0.15 mmol and (*d*) 0.2 mmol of [Pb(S_2_COOct)_2_].

**Figure 6.**
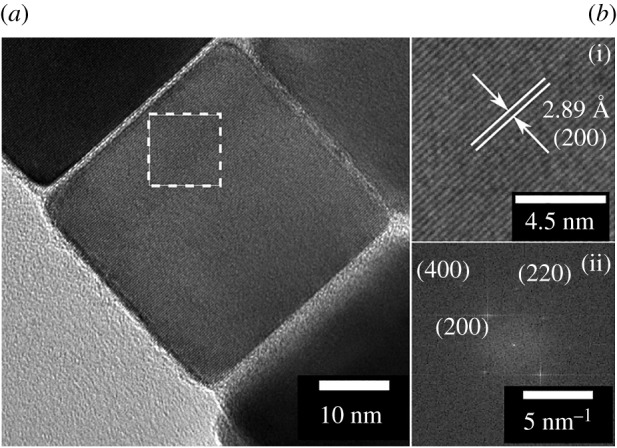
(*a*) HRTEM image of a PbS nanocrystal extracted from the film using 0.2 mmol of [Pb(S_2_COOct)_2_]. (*b*(i)) Expanded image from the white box region showing the 〈200〉 plane of PbS (galena) and (*b*(ii)) fast Fourier transform of the selected region.

TEM of PbS nanocrystals extracted from the sulfur polymer films followed by dissolution in chloroform showed well-defined cubes with occasional slight elongation forming irregular cuboids (figure 5*a*–*d*). The nanocrystal size showed no trend with increasing concentration although all nanocrystals produced were under 80 nm in edge length. When using 0.1 mmol (60 mg) of [Pb(S_2_COOct)_2_] the nanocrystals formed showed the greatest monodispersity and lowest mean size (28.6 nm) (figure 5*b*). This suggests that 0.1 mmol of [Pb(S_2_COOct)_2_] is the optimal amount of precursor quantity in terms of particle monodispersity. HRTEM also confirmed that the cubes were PbS by lattice fringes showing (200) and fast Fourier transform showing (200), (220) and (400) reflections, figure 6, which was further supported by selected area electron diffraction (SAED) (see electronic supplementary material, figure S16).

### 3.5. Optical characterization

**Figure 7.**
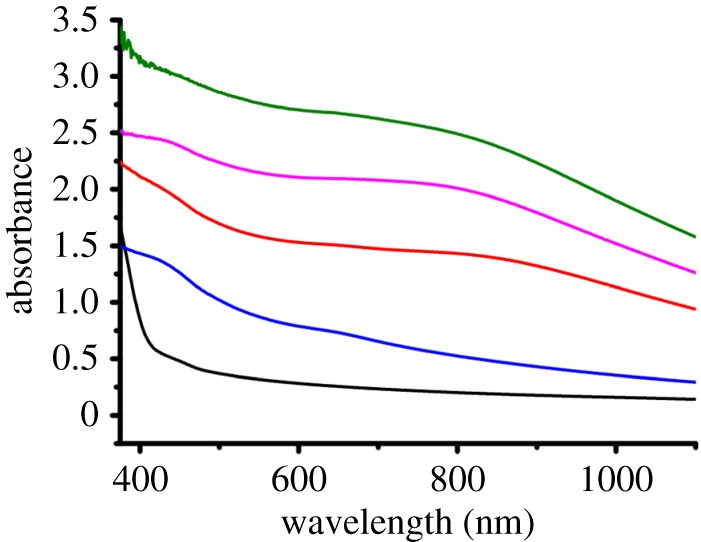
UV/visible spectra of films containing different concentrations of [Pb(S_2_COOct)_2_]: 0.1 mmol in polymer (**4**) prior to decomposition (black), and after heating 0.05 mmol (blue), 0.1 mmol (red), 0.15 mmol (pink) and 0.2 mmol (green).

UV/visible spectra of the films allowed observation of the concentration of nanocrystals within the films upon variation of [Pb(S_2_COOct)_2_]:S-polymer (polymer (**4**)) concentration ratios (figure 7). All films exhibited broad absorption over the entire visible range, which is typical of PbS (see electronic supplementary material, figure S17). The broad absorption increased between samples of polymer (**3**), an unreacted film of polymer (**3**) containing [Pb(S_2_COOct)_2_] and film of polymer (**3**) containing [Pb(S_2_COOct)_2_] heated at 150°C under nitrogen for 15 min. Introduction of the PbS nanocrystals generally increases the absorption of the thin films across the visible as expected due to the intense black colour of PbS demonstrating the ability to use the PbS embedded S-polymer films as absorptive optical filters.

## 4. Conclusion

Films of a solution processable sulfur polymer containing networks of PbS nanocrystals have been produced via spin coating of sulfur polymers with lead(II) *n*-octylxanthate, [Pb(S_2_COOct)_2_], and the subsequent heating of the films. The solubility of the sulfur polymer was optimized for solution processing by the addition of bisphenol A dimethacrylate and 1,3-diisopropenylbenzene to molten elemental sulfur in an ‘inverse vulcanization’ process to form poly(sulfur-*co*-bisphenol A dimethacrylate-*co*-1,3-diisopropenylbenzene). Upon heating films of sulfur polymer and lead(II) *n*-octylxanthate, PbS nanocrystals were found to form within the sulfur polymer film. Monitoring the formation of PbS with XPS and XRD showed the optimum temperature for the breakdown of [Pb(S_2_COOct)_2_] was 150°C. The influence of the ratio of sulfur polymer to [Pb(S_2_COOct)_2_] on the morphology of the films showed that a limit of PbS nanocrystals within the polymer was passed when 0.05 mmol of [Pb(S_2_COOct)_2_] was added resulting in a 79.8 wt% film of PbS in polymer. Above this value ejection of PbS nanocrystals from the film occurred causing formation of nanocrystals on the surface. The edge length and monodispersity of the PbS nanocrystals showed a minimum when 0.1 mmol of [Pb(S_2_COOct)_2_] was used. The thin films produced allow the growth of PbS nanocrystals within the S-polymer distributed in the film similar to our previous report when using lead(II) alkylxanthates and polystyrene. The development of a solution processable process to thin films of sulfur-based polymers containing highly optically absorbent inorganic nanocrystals demonstrates the potential for the use of sulfur-based polymers as an alternative to traditional polymers for absorbent optical filters, or colour gels.

## Supplementary Material

Electronic Supporting Information

## Data accessibility

The additional XPS spectra, XRD patterns, DSC curves, NMR spectra, EDS spectra, SAED patterns, photographs and UV–visible spectra referenced in the text are included in the electronic supplementary material.

## References

[1] Rath T, Trimmel G 2013 *In situ* syntheses of semiconducting nanoparticles in conjugated polymer matrices and their application in photovoltaics. Hybrid Mater. 1, 15–36. (doi:10.2478/hyma-2013-0003)

[2] Lewis EA et al. 2015 *In situ* synthesis of PbS nanocrystals in polymer thin films from lead(II) xanthate and dithiocarbamate complexes: evidence for size and morphology control. Chem. Mater. 27, 2127–2136. (doi:10.1021/cm504765z)

[3] MacLachlan AJ et al. 2015 Polymer/nanocrystal hybrid solar cells: influence of molecular precursor design on film nanomorphology, charge generation and device performance. Adv. Funct. Mater. 25, 409–420. (doi:10.1002/adfm.201403108) 25866496 10.1002/adfm.201403108 PMC4384757

[4] Bansal AK et al. 2014 Photophysical and structural characterisation of *in situ* formed quantum dots. Phys. Chem. Chem. Phys. 16, 9556–9564. (doi:10.1039/C4CP00727A) 24727793 10.1039/c4cp00727a

[5] Agrawal V, Jain K, Arora L, Chand S 2013 Synthesis of CdS nanocrystals in poly(3-hexylthiophene) polymer matrix: optical and structural studies. J. Nanoparticle Res. 15, 1–14. (doi:10.1007/s11051-013-1697-z)

[6] Dowland S, Lutz T, Ward A, King SP, Sudlow A, Hill MS, Molloy KC, Haque SA 2011 Direct growth of metal sulfide nanoparticle networks in solid-state polymer films for hybrid inorganic–organic solar cells. Adv. Mater. 23, 2739–2744. (doi:10.1002/adma.201100625) 21520466 10.1002/adma.201100625

[7] Leventis HC, King SP, Sudlow A, Hill MS, Molloy KC, Haque SA 2010 Nanostructured hybrid polymer−inorganic solar cell active layers formed by controllable *in situ* growth of semiconducting sulfide networks. Nano Lett. 10, 1253–1258. (doi:10.1021/nl903787j) 20225884 10.1021/nl903787j

[8] Kaltenhauser V et al. 2013 Bismuth sulphide– polymer nanocomposites from a highly soluble bismuth xanthate precursor. J. Mater. Chem. C 1, 7825–7832. (doi:10.1039/C3TC31684J)

[9] Fradler C, Rath T, Dunst S, Letofsky-Papst I, Saf R, Kunert B, Hofer F, Resel R, Trimmel G 2014 Flexible polymer/copper indium sulfide hybrid solar cells and modules based on the metal xanthate route and low temperature annealing. Sol. Energy Mater. Sol. Cells 124, 117–125. (doi:10.1016/j.solmat.2014.01.043)

[10] Rath T et al. 2011 A direct route towards polymer/copper indium sulfide nanocomposite solar cells. Adv. Energy Mater. 1, 1046–1050. (doi:10.1002/aenm.201100442)

[11] Bansal N, O'Mahony FTF, Lutz T, Haque SA 2013 Solution processed polymer–inorganic semiconductor solar cells employing Sb_2_S_3_ as a light harvesting and electron transporting material. Adv. Energy Mater. 3, 986–990. (doi:10.1002/aenm.201300017)

[12] Luther JM, Law M, Beard MC, Song Q, Reese MO, Ellingson RJ, Nozik AJ 2008 Schottky solar cells based on colloidal nanocrystal films. Nano Lett. 8, 3488–3492. (doi:10.1021/nl802476m) 18729414 10.1021/nl802476m

[13] Yan J, McNaughter PD, Wang Z, Hodson N, Chen M, Cui Z, O'Brien P, Saunders BR 2015 Controlled aggregation of quantum dot dispersions by added amine bilinkers and effects on hybrid polymer film properties. RSC Adv. 5, 95 512–95 522. (doi:10.1039/C5RA15009D)

[14] Saunders BR, Turner ML 2008 Nanoparticle– polymer photovoltaic cells. Adv. Colloid Interface 138, 1–23. (doi:10.1016/j.cis.2007.09.001) 10.1016/j.cis.2007.09.001 17976501

[15] Matthews PD, McNaughter PD, Lewis DJ, O'Brien P 2017 Shining a light on transition metal chalcogenides for sustainable photovoltaics. Chem. Sci. 8, 4177–4187. (doi:10.1039/C7SC00642J) 28626562 10.1039/c7sc00642j PMC5468987

[16] Crick CR, Bear JC, Southern P, Parkin IP 2013 A general method for the incorporation of nanoparticles into superhydrophobic films by aerosol assisted chemical vapour deposition. J. Mater. Chem. A 1, 4336–4344. (doi:10.1039/C3TA01629C)

[17] Rupprechter G, Hayek K, Hofmeister H 1998 Electron microscopy of thin-film model catalysts: activation of alumina-supported rhodium nanoparticles. J. Catal. 173, 409–422. (doi:10.1006/jcat.1997.1917)

[18] Costa NJS, Rossi LM 2012 Synthesis of supported metal nanoparticle catalysts using ligand assisted methods. Nanoscale 4, 5826–5834. (doi:10.1039/c2nr31165h) 22915064 10.1039/c2nr31165h

[19] Lee D, Rubner MF, Cohen RE 2006 All-nanoparticle thin-film coatings. Nano Lett. 6, 2305–2312. (doi:10.1021/nl061776m) 17034102 10.1021/nl061776m

[20] Tu Y, Zhou L, Jin YZ, Gao C, Ye ZZ, Yang YF, Wang QL 2010 Transparent and flexible thin films of ZnO-polystyrene nanocomposite for UV-shielding applications. J. Mater. Chem. 20, 1594–1599. (doi:10.1039/B914156A)

[21] Zou G, Ju H 2004 Electrogenerated chemiluminescence from a CdSe nanocrystal film and its sensing application in aqueous solution. Anal. Chem. 76, 6871–6876. (doi:10.1021/ac049012j) 15571335 10.1021/ac049012j

[22] Van Toan N, Chien NV, Van Duy N, Vuong DD, Lam NH, Hoa ND, Van Hieu N, Chien ND 2015 Scalable fabrication of SnO_2_ thin films sensitized with CuO islands for enhanced H_2_S gas sensing performance. Appl. Surf. Sci. 324, 280–285. (doi:10.1016/j.apsusc.2014.10.134)

[23] Tao W-H, Tsai C-H 2002 H_2_S sensing properties of noble metal doped WO_3_ thin film sensor fabricated by micromachining. Sens. Actuators B Chem. 81, 237–247. (doi:10.1016/S0925-4005(01)00958-3)

[24] Sadasivuni KK, Kafy A, Zhai L, Ko H-U, Mun S, Kim J 2015 Transparent and flexible cellulose nanocrystal/reduced graphene oxide film for proximity sensing. Small 11, 994–1002. (doi:10.1002/smll.201402109) 25293649 10.1002/smll.201402109

[25] Shukla S, Seal S, Ludwig L, Parish C 2004 Nanocrystalline indium oxide-doped tin oxide thin film as low temperature hydrogen sensor. Sens. Actuators B Chem. 97, 256–265. (doi:10.1016/j.snb.2003.08.025)

[26] Crick CR, Bear JC, Kafizas A, Parkin IP 2012 Superhydrophobic photocatalytic surfaces through direct incorporation of titania nanoparticles into a polymer matrix by aerosol assisted chemical vapor deposition. Adv. Mater. 24, 3505–3508. (doi:10.1002/adma.201201239) 22706974 10.1002/adma.201201239

[27] Page K, Palgrave RG, Parkin IP, Wilson M, Savin SLP, Chadwick AV 2006 Titania and silver–titania composite films on glass—potent antimicrobial coatings. J. Mater. Chem. 17, 95–104. (doi:10.1039/B611740F)

[28] Noimark S, Page K, Bear JC, Sotelo-Vazquez C, Quesada-Cabrera R, Lu Y, Allan E, Darr JA, Parkin IP 2015 Functionalised gold and titania nanoparticles and surfaces for use as antimicrobial coatings. Faraday Discuss. 175, 273–287. (doi:10.1039/C4FD00113C) 10.1039/c4fd00113c 25370309

[29] Walters G, Parkin IP 2009 The incorporation of noble metal nanoparticles into host matrix thin films: synthesis, characterisation and applications. J. Mater. Chem. 19, 574–590. (doi:10.1039/B809646E)

[30] Hogarth G 2005 Transition metal dithiocarbamates: 1978–2003. In Progress in inorganic chemistry, vol. 53 (ed. KD Karlin), pp. 71–561. Hoboken, NJ: Wiley (doi:10.1002/0471725587.ch2)

[31] Hollingsworth N et al. 2014 The active nature of primary amines during the thermal decomposition of nickel-dithiocarbamates to nickel-sulfide nanoparticles. Chem. Mater. 26, 6281–6292. (doi:10.1021/cm503174z)

[32] Boadi NO, Malik MA, O'Brien P, Awudza JAM 2012 Single source molecular precursor routes to lead chalcogenides. Dalton Trans. 41, 10 497–10 506. (doi:10.1039/C2DT30849E) 10.1039/c2dt30849e 22791196

[33] Trindade T, O'Brien P 1996 Synthesis of CdS and CdSe nanoparticles by thermolysis of diethyldithio-or diethyldiseleno-carbamates of cadmium. J. Mater. Chem. 6, 343–347. (doi:10.1039/JM9960600343)

[34] Malik MA, Revaprasadu N, O'Brien P 2001 Air-stable single-source precursors for the synthesis of chalcogenide semiconductor nanoparticles. Chem. Mater. 13, 913–920. (doi:10.1021/cm0011662)

[35] Nair PS, Radhakrishnan T, Revaprasadu N, Kolawole GA, O'Brien P 2002 A single-source route to CdS nanorods. Chem. Commun. 564–565. (doi:10.1039/B200434H) 10.1039/b200434h 12120125

[36] Brent JR, McNaughter PD, O'Brien P 2017 Precursor determined lateral size control of monolayer MoS_2_ nanosheets from a series of alkylammonium thiomolybdates: a reversal of trend between growth media. Chem. Commun. 53, 6428–6431. (doi:10.1039/C7CC01641G) 10.1039/c7cc01641g 28560371

[37] Clark JM, Kociok-Köhn G, Harnett NJ, Hill MS, Hill R, Molloy KC, Saponia H, Stanton D, Sudlow A 2011 Formation of PbS materials from lead xanthate precursors. Dalton Trans. 40, 6893–6900. (doi:10.1039/C1DT10273G) 21647494 10.1039/c1dt10273g

[38] Akhtar J, Afzaal M, Vincent MA, Burton NA, Hillier IH, O'Brien P 2011 Low temperature CVD growth of PbS films on plastic substrates. Chem. Commun. 47, 1991–1993. (doi:10.1039/C0CC05036A) 10.1039/c0cc05036a 21234486

[39] McNaughter PD, Saah SA, Akhtar M, Abdulwahab K, Malik MA, Raftery J, Awudza JAM, O'Brien P 2016 The effect of alkyl chain length on the structure of lead(II) xanthates and their decomposition to PbS in melt reactions. Dalton Trans. 45, 16 345–16 353. (doi:10.1039/c6dt02859d) 10.1039/c6dt02859d 27722337

[40] Al-Shakban M, Matthews PD, Deogratias G, McNaughter PD, Raftery J, Vitorica-Yrezabal I, Mubofu EB, O'Brien P 2017 Novel xanthate complexes for the size-controlled synthesis of copper sulfide nanorods. Inorg. Chem. 56, 9247–9254. (doi:10.1021/acs.inorgchem.7b01288) 28722401 10.1021/acs.inorgchem.7b01288

[41] Afzaal M, Ellwood K, Pickett NL, O'Brien P, Raftery J, Waters J 2004 Growth of lead chalcogenide thin films using single-source precursors. J. Mater. Chem. 14, 1310–1315. (doi:10.1039/B313063K)

[42] Akhtar J, Akhtar M, Malik MA, O'Brien P, Raftery J 2012 A Single-source precursor route to unusual PbSe nanostructures by a solution–liquid–solid method. J. Am. Chem. Soc. 134, 2485–2487. (doi:10.1021/ja209831n) 22280503 10.1021/ja209831n

[43] Trindade T, Monteiro OC, O'Brien P, Motevalli M 1999 Synthesis of PbSe nanocrystallites using a single-source method. The X-ray crystal structure of lead (II) diethyldiselenocarbamate. Polyhedron 18, 1171–1175. (doi:10.1016/S0277-5387(98)00411-2)

[44] Akhtar J, Malik MA, Stubbs SK, O'Brien P, Helliwell M, Binks DJ 2011 Morphology-tailored synthesis of PbSe nanocrystals and thin films from bis[N,N-diisobutyl-N′-(4-nitrobenzoyl) selenoureato]lead(II). Eur. J. Inorg. Chem. 2011, 2984–2990. (doi:10.1002/ejic.201100190)

[45] Nguyen CQ, Adeogun A, Afzaal M, Malik MA, O'Brien P 2006 Metal complexes of selenophosphinates from reactions with (R_2_PSe)_2_Se: [M(R_2_PSe_2_)_n_] (M = Zn^II^, Cd^II^, Pb^II^, In^III^, Ga^III^, Cu^I^, Bi^III^, Ni^II^; R = ^i^Pr, Ph) and [MoV_2_O_2_Se_2_(Se_2_PiPr_2_)_2_]. Chem. Commun. 2182–2184. (doi:10.1039/B603198F) 10.1039/b603198f 16703148

[46] Duan T, Lou W, Wang X, Xue Q 2007 Size-controlled synthesis of orderly organized cube-shaped lead sulfide nanocrystals via a solvothermal single-source precursor method. Colloids Surf. A 310, 86–93. (doi:10.1016/j.colsurfa.2007.06.006)

[47] Ritch JS, Chivers T, Ahmad K, Afzaal M, O'Brien P 2010 Synthesis, structures, and multinuclear NMR spectra of tin(II) and lead(II) complexes of tellurium-containing imidodiphosphinate ligands: preparation of two morphologies of phase-pure PbTe from a single-source precursor. Inorg. Chem. 49, 1198–1205. (doi:10.1021/ic9021728) 20041664 10.1021/ic9021728

[48] Boadi NO, McNaughter PD, Helliwell M, Malik MA, Awudza JAM, O'Brien P 2016 The deposition of PbS and PbSe thin films from lead dichalcogenoimidophosphinates by AACVD. Inorg. Chim. Acta 453, 439–442. (doi:10.1016/j.ica.2016.08.023)

[49] Kutney G 2013 Sulfur: history, technology, applications and industry, 2nd edn Toronto, Canada: ChemTec Publishing.

[50] Bear JC, Peveler WJ, McNaughter PD, Parkin IP, O'Brien P, Dunnill CW 2015 Nanoparticle–sulphur ‘inverse vulcanisation’ polymer composites. Chem. Commun. 51, 10 467–10 470. (doi:10.1039/C5CC03419A) 10.1039/c5cc03419a 26028319

[51] Knight L 2014 Sulphur surplus: up to our necks in a diabolical element. *BBC News*. See http://www.bbc.co.uk/news/magazine-28369829 (accessed 27 May 2016).

[52] Lim J, Pyun J, Char K 2015 Recent approaches for the direct use of elemental sulfur in the synthesis and processing of advanced materials. Angew. Chem. Int. Ed. 54, 3249–3258. (doi:10.1002/anie.201409468) 10.1002/anie.201409468 25583026

[53] Priegert AM, Rawe BW, Serin SC, Gates DP 2016 Polymers and the p-block elements. Chem. Soc. Rev. 45, 922–953. (doi:10.1039/C5CS00725A) 26524670 10.1039/c5cs00725a

[54] Griebel JJ, Glass RS, Char K, Pyun J 2016 Polymerizations with elemental sulfur: a novel route to high sulfur content polymers for sustainability, energy and defense. Prog. Polym. Sci. 58, 90–125. (doi:10.1016/j.progpolymsci.2016.04.003)

[55] Simmonds AG et al. 2014 Inverse vulcanization of elemental sulfur to prepare polymeric electrode materials for Li–S batteries. ACS Macro Lett. 3, 229–232. (doi:10.1021/mz400649w) 10.1021/mz400649w 35590512

[56] Griebel JJ, Li G, Glass RS, Char K, Pyun J 2015 Kilogram scale inverse vulcanization of elemental sulfur to prepare high capacity polymer electrodes for Li-S batteries. J. Polym. Sci. Part A: Polym. Chem. 53, 173–177. (doi:10.1002/pola.27314)

[57] Griebel JJ et al. 2014 New infrared transmitting material via inverse vulcanization of elemental sulfur to prepare high refractive index polymers. Adv. Mater. 26, 3014–3018. (doi:10.1002/adma.201305607) 24659231 10.1002/adma.201305607

[58] Griebel JJ, Nguyen NA, Namnabat S, Anderson LE, Glass RS, Norwood RA, Mackay ME, Char K, Pyun J 2015 Dynamic covalent polymers via inverse vulcanization of elemental sulfur for healable infrared optical materials. ACS Macro Lett. 4, 862–866. (doi:10.1021/acsmacrolett.5b00502) 10.1021/acsmacrolett.5b00502 35596448

[59] Hasell T, Parker DJ, Jones HA, McAllister T, Howdle SM. 2016 Porous inverse vulcanised polymers for mercury capture. Chem. Commun. 52, 5383–5386. (doi:10.1039/C6CC00938G) 10.1039/c6cc00938g 26931278

[60] Crockett MP et al. 2016 Sulfur-limonene polysulfide: a material synthesized entirely from industrial by-products and its use in removing toxic metals from water and soil. Angew. Chem. Int. Ed. 55, 1714–1718. (doi:10.1002/anie.201508708) 10.1002/anie.201508708 PMC4755153 26481099

[61] Bear JC, McGettrick JD, Parkin IP, Dunnill CW, Hasell T 2016 Porous carbons from inverse vulcanised polymers. Micropor. Mesopor. Mater. 232, 189–195. (doi:10.1016/j.micromeso.2016.06.021)

[62] Chung WJ et al. 2013 The use of elemental sulfur as an alternative feedstock for polymeric materials. Nat. Chem. 5, 518–524. (doi:10.1038/nchem.1624) 23695634 10.1038/nchem.1624

[63] Wei Y, Li X, Xu Z, Sun H, Zheng Y, Peng L, Liu Z, Gao C, Gao M 2015 Solution processible hyperbranched inverse-vulcanized polymers as new cathode materials in Li-S batteries. Polym. Chem. 6, 973–982. (doi:10.1039/C4PY01055H)

[64] Jolanki R, Kanerva L, Estlander T 1995 Occupational allergic contact dermatitis caused by epoxy diacrylate in ultraviolet-light-cured paint, and bisphenol A in dental composite resin. Contact Dermatitis 33, 94–99. (doi:10.1111/j.1600-0536.1995.tb00508.x) 8549151 10.1111/j.1600-0536.1995.tb00508.x

[65] Meyer B 1976 Elemental sulfur. Chem. Rev. 76, 367–388. (doi:10.1021/cr60301a003)

[66] Tse JS, Klug DD 1999 Structure and dynamics of liquid sulphur. Phys. Rev. B 59, 34–37. (doi:10.1103/PhysRevB.59.34)

[67] Salman MK, Karabay B, Karabay LC, Cihaner A. 2016 Elemental sulfur-based polymeric materials: synthesis and characterization. J. Appl. Polym. Sci. 133, 43655 (doi:10.1002/app.43655)

[68] Arslan M, Kiskan B, Yagci Y 2016 Combining elemental sulfur with polybenzoxazines via inverse vulcanization. Macromolecules 49, 767–773. (doi:10.1021/acs.macromol.5b02791)

[69] Parker DJ, Jones HA, Petcher S, Cervini L, Griffin JM, Akhtar R, Hasell T 2017 Low cost and renewable sulfur-polymers by inverse vulcanisation, and their potential for mercury capture. J. Mater. Chem. A 5, 11 682–11 692. (doi:10.1039/C6TA09862B)

[70] Muscat J, Klauber C 2001 A combined ab initio and photoelectron study of galena (PbS). Surf. Sci. 491, 226–238. (doi:10.1016/S0039-6028(01)01408-X)

[71] Thomas PJ et al. 2015 Growth of nanocrystalline thin films of metal sulfides [CdS, ZnS, CuS and PbS] at the water–oil interface. RSC Adv. 5, 62 291–62 299. (doi:10.1039/C5RA09417H)

[72] McFeely FR, Kowalczyk S, Ley L, Pollak RA, Shirley DA 1973 High-resolution X-ray-photoemission spectra of PbS, PbSe, and PbTe valence bands. Phys. Rev. B 7, 5228–5237. (doi:10.1103/PhysRevB.7.5228)

[73] Kartio I, Laajalehto K, Suoninen E 1999 Characterization of the ethyl xanthate adsorption layer on galena (PbS) by synchrotron radiation excited photoelectron spectroscopy. Colloids Surf. A 154, 97–101. (doi:10.1016/S0927-7757(98)00912-1)

[74] Cant DJH et al. 2015 Surface properties of nanocrystalline PbS films deposited at the water–oil interface: a study of atmospheric aging. Langmuir 31, 1445–1453. (doi:10.1021/la504779h) 25557338 10.1021/la504779h

[75] NIST X-ray Photoelectron Spectroscopy (XPS) Database, version 3.5. See http://srdata.nist.gov/xps/ (accessed 23 February 2015).

[76] Buckley AN, Woods R 1984 An X-ray photoelectron spectroscopic study of the oxidation of galena. Appl. Surf. Sci. 17, 401–414. (doi:10.1016/0378-5963(84)90003-5)

[77] Moulder JF, Stickle WF, Sobol PE, Bomben KD. 1995 Handbook of X ray photoelectron spectroscopy: a reference book of standard spectra for identification and interpretation of XPS data. Eden Prairie, MN: Physical Electronics.

