# Supplementary material for: The in situ synthesis of PbS nanocrystals from lead(II) n-octylxanthate within a 1,3-diisopropenylbenzene–bisphenol A dimethacrylate sulfur copolymer
Source: R Soc Open Sci. 2017 Aug 16;4(8):170383. doi: 10.1098/rsos.170383 (PMC5579102; doi:10.1098/rsos.170383)
Supplement: Electronic Supporting Information [file rsos170383supp1.docx]

The *in situ* synthesis of PbS nanocrystals from lead(II) *n*-octylxanthate within a 1,3-diisopropenyl benzene-bisphenol A dimethacrylate sulfur copolymer-Supporting Information

P. D. McNaughter,^a,†^ J. C. Bear,^b,†^ A. G. Mayes,^c^ I. P. Parkin^b,^* and P. O’Brien^a,d,^*

a. School of Chemistry, The University of Manchester, Oxford Road, Manchester, M13 9PL, United Kingdom.

b. Materials Chemistry Centre, Department of Chemistry, University College London, 20 Gordon Street, London, WC1H 0AJ, UK.

c. School of Chemistry, University of East Anglia, Norwich Research Park, Norwich, Norfolk. NR4 7TJ, United Kingdom.

d. School of Materials, The University of Manchester, Oxford Road, Manchester, M13 9PL, United Kingdom.

† Authors contributed equally


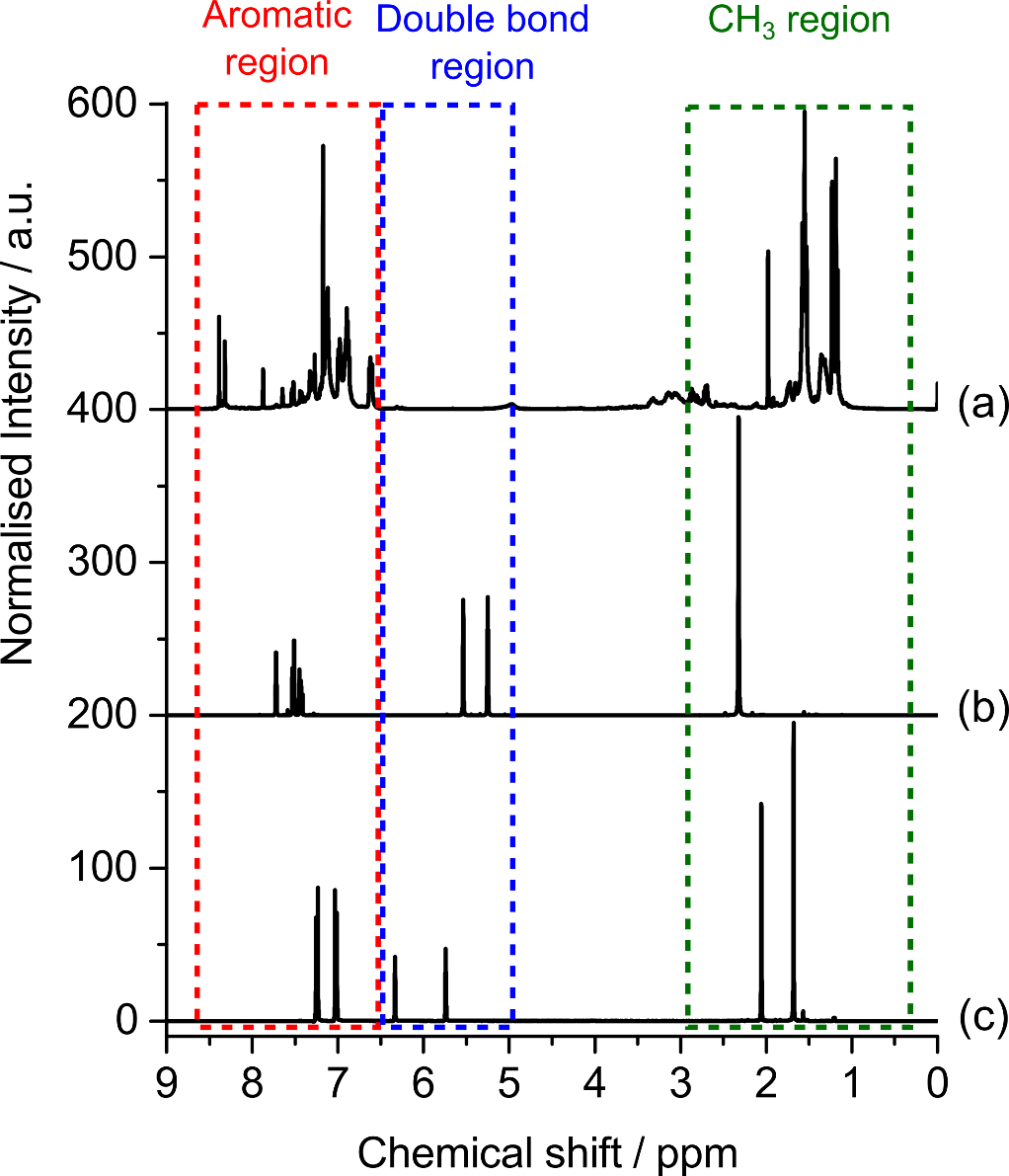


**Figure S1**: Nuclear Magnetic Resonance spectra of (a) a typical poly(sulfur-*co*-bisphenol A dimethacrylate-*co*-1,3-diisopropenylbenzene), (b) 1,3-diisopropenylbenzene and (c) bisphenol A dimethacrylate.


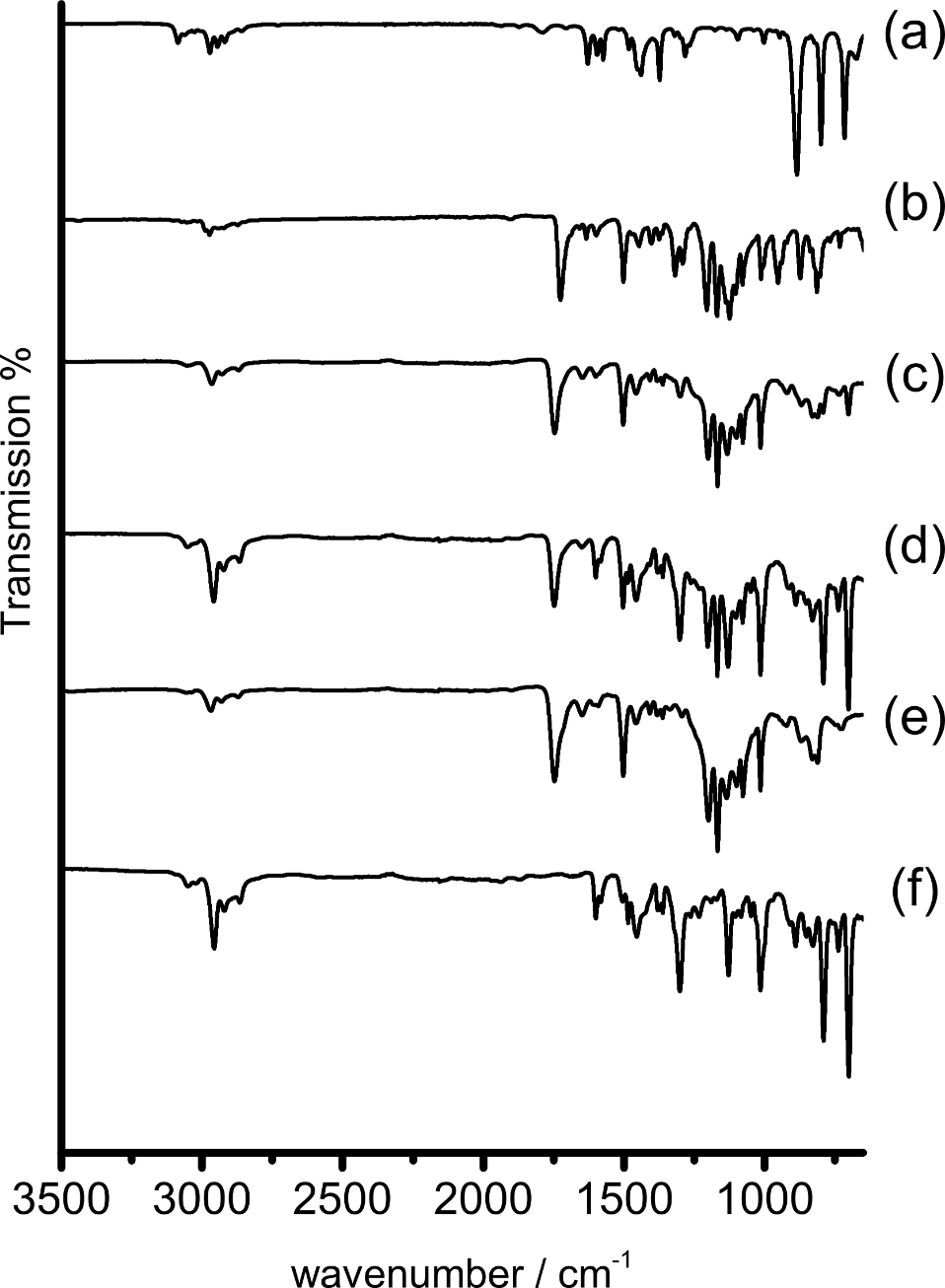


**Figure S2:** FT-IR spectra of (a) 1,3-diisopropenylbenzene, (b) bisphenol A dimethacrylate,

(c) poly(sulfur-*co*-bisphenol A dimethacrylate-*co*-1,3-diisopropenylbenzene) (**3**), (d) poly(sulfur-*co*-bisphenol A dimethacrylate-*co*-1,3-diisopropenylbenzene) (**4**), (e) poly(sulfur-*co*-bisphenol A dimethacrylate) (**2**) and (f) poly(sulfur-*co*-1,3-diisopropenylbenzene) (**1**).


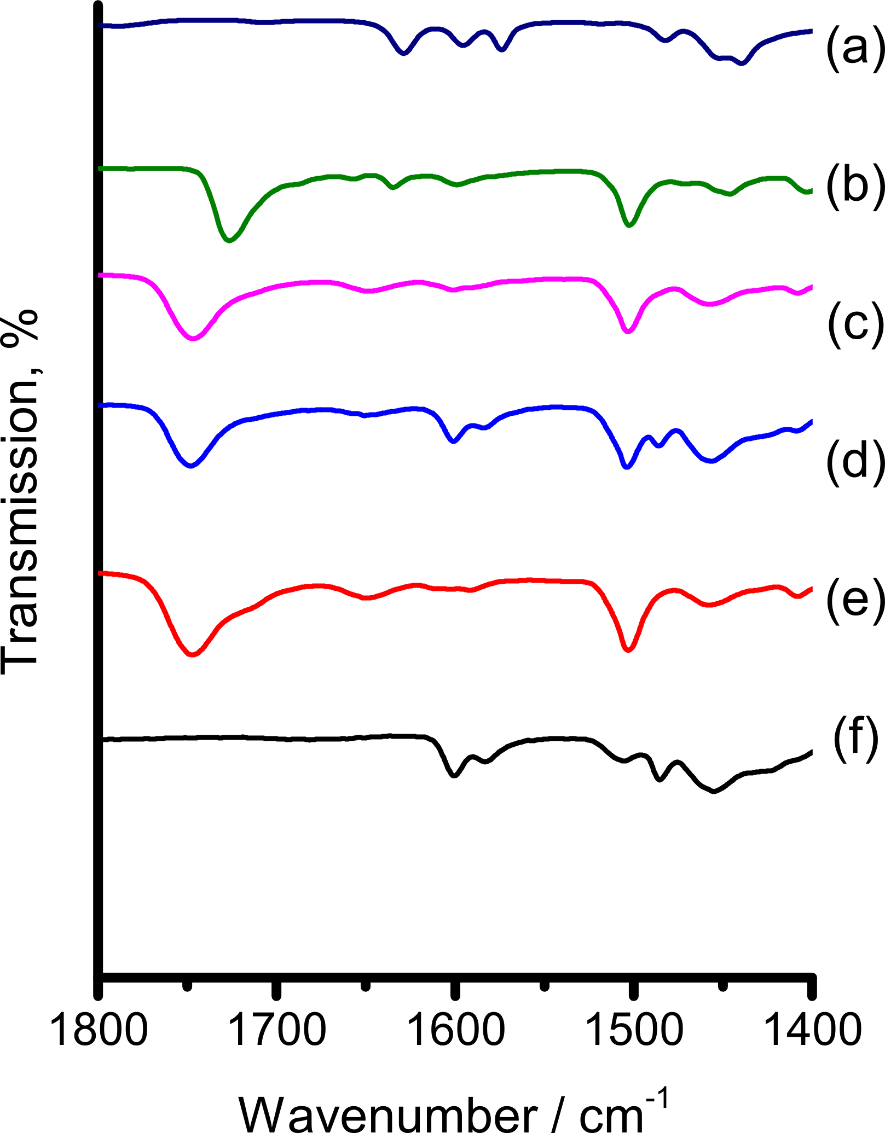


**Figure S3:** FT-IR spectra expansion on the C=O and C=C regions for (a) 1,3-diisopropenylbenzene, (b) bisphenol A dimethacrylate,

(c) poly(sulfur-*co*-bisphenol A dimethacrylate-*co*-1,3-diisopropenylbenzene) (**3**), (d) poly(sulfur-*co*-bisphenol A dimethacrylate-*co*-1,3-diisopropenylbenzene) (**4**), (e) poly(sulfur-*co*-bisphenol A dimethacrylate) (**2**) and (f) poly(sulfur-*co*-1,3-diisopropenylbenzene) (**1**).


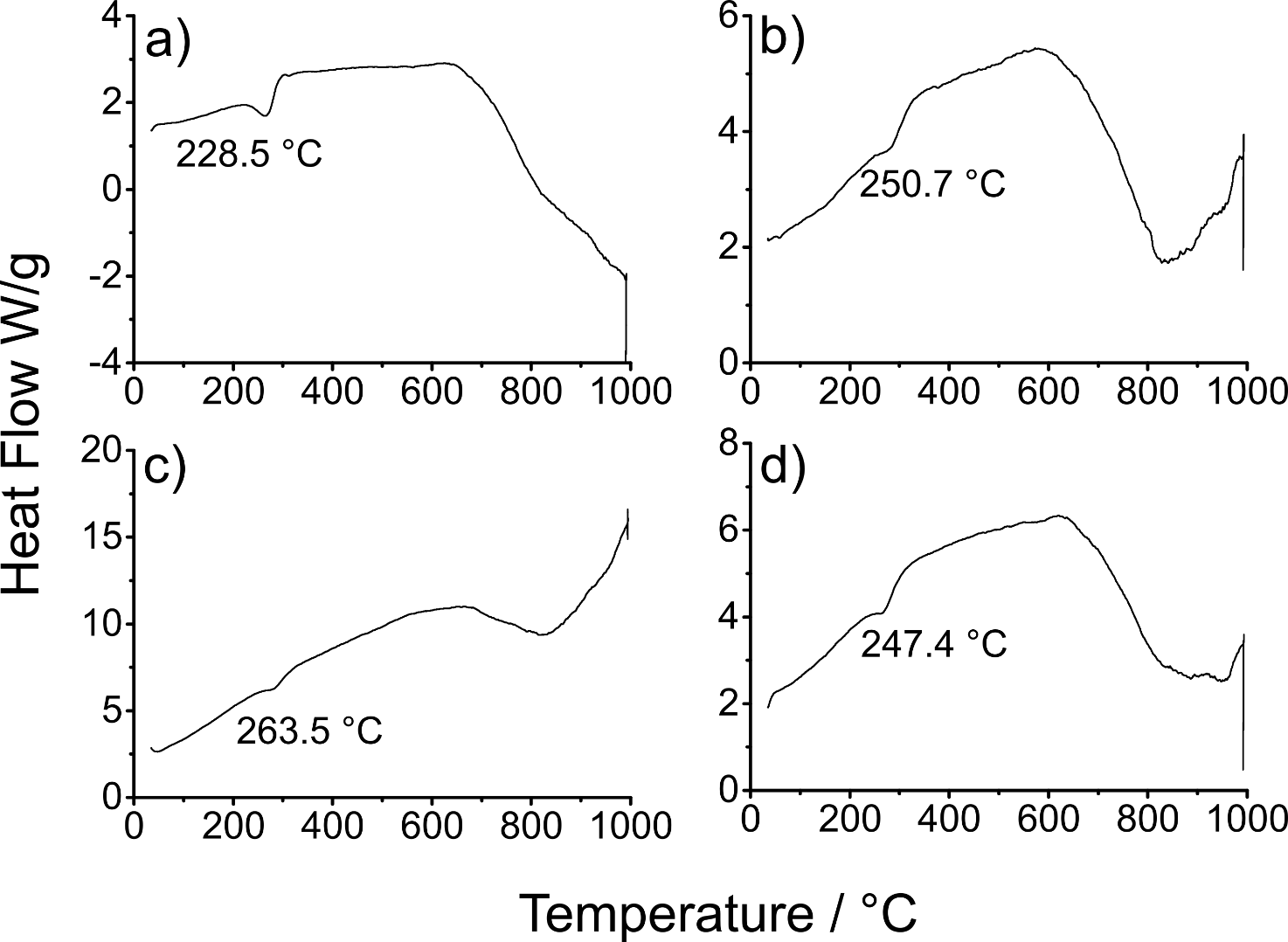


**Figure S4**: Differential Scanning Calorimetry heat flow vs. Temperature curves for: a) polymer (**1**) ((poly(sulfur-*co*-1,3-diisopropenylbenzene), b) polymer (**2**) (poly(sulfur-*co*-bisphenol A dimethacrylate), c) polymer (**3**) (poly(sulfur-*co*-bisphenol A dimethacrylate-*co*-1,3-diisopropenylbenzene), higher ratio of 1,3-diisopropenylbenzene) and d) (poly(sulfur-*co*-bisphenol A dimethacrylate-*co*-1,3-diisopropenylbenzene), lower ratio of 1,3-diisopropenylbenzene), showing the onset points of decomposition.


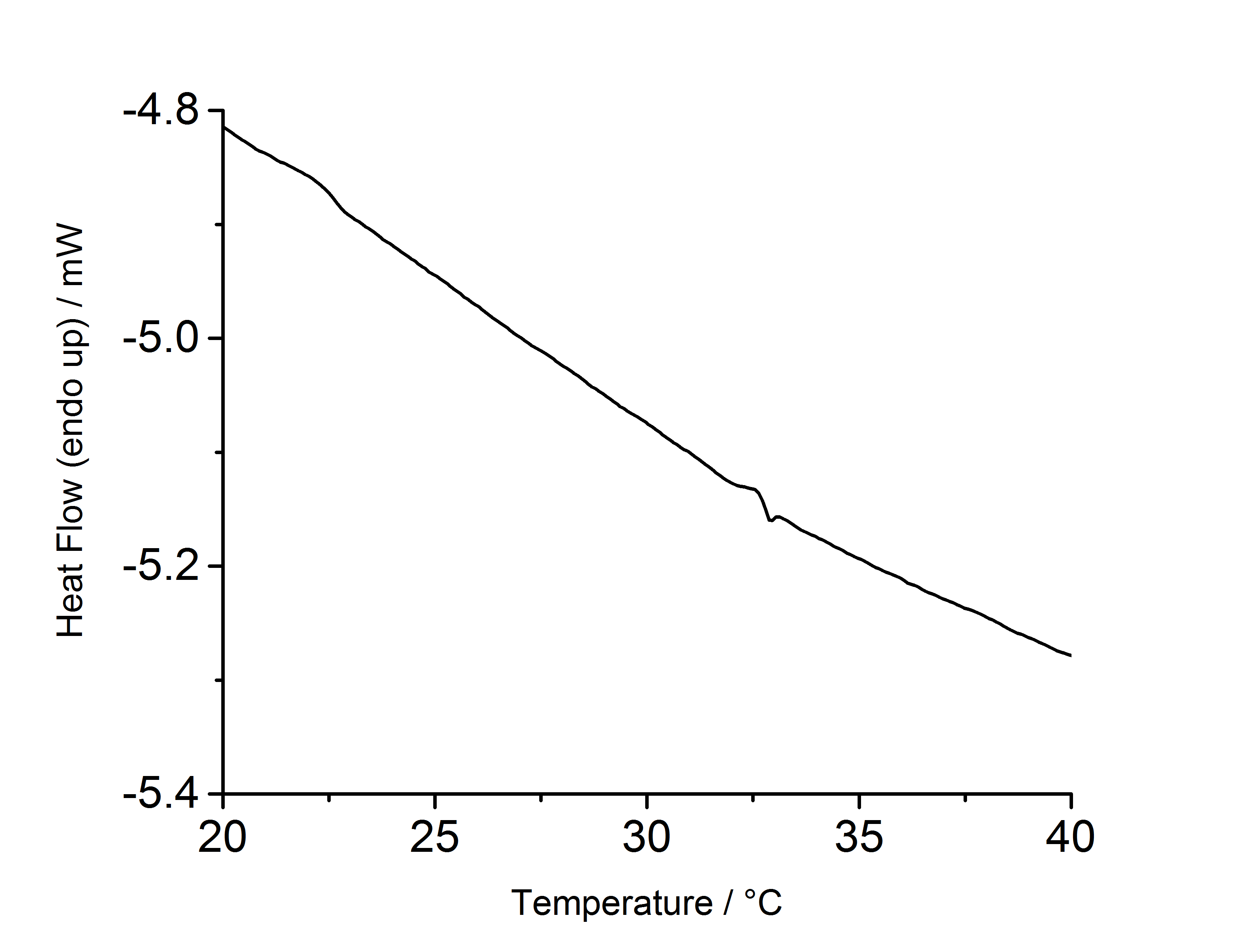


**Figure S5**: Differential Scanning Calorimetry heat flow vs. Temperature cooling curve for polymer (**1**) ((poly(sulfur-*co*-1,3-diisopropenylbenzene showing the T_g_ onset at 32.2 °C.


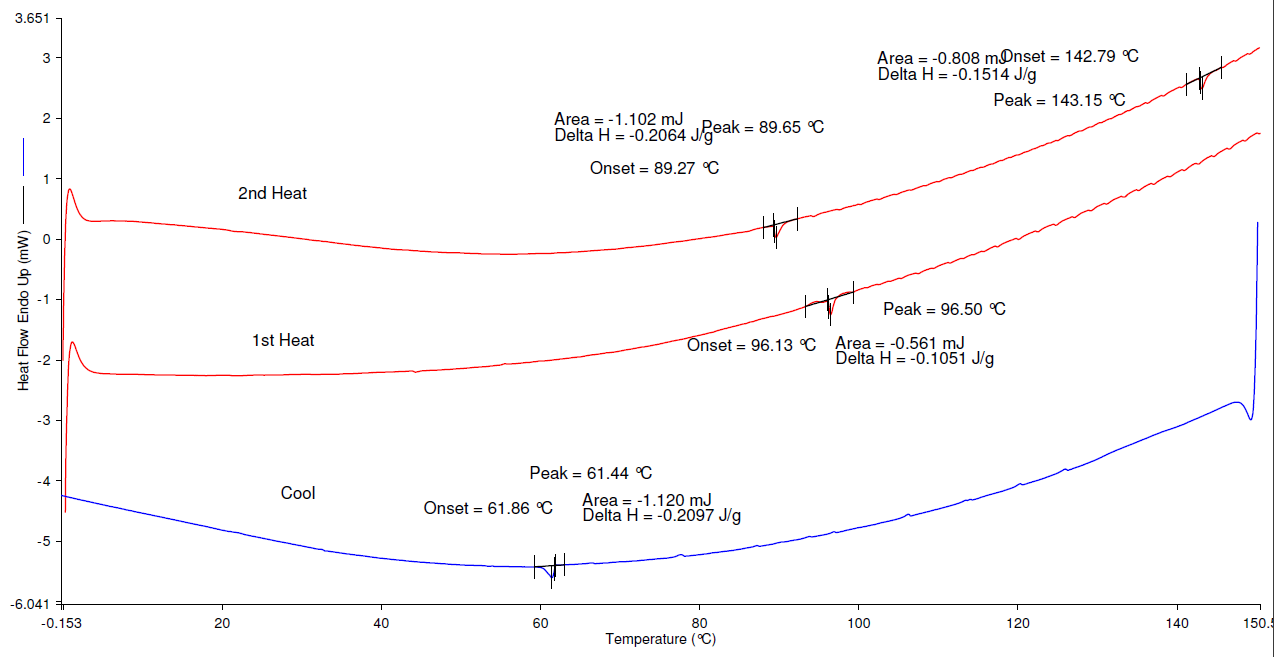


**Figure S6**: Differential Scanning Calorimetry heat flow vs. Temperature curves for polymer (**1**) ((poly(sulfur-*co*-1,3-diisopropenylbenzene with heating curves (red) and cooling curve (blue).


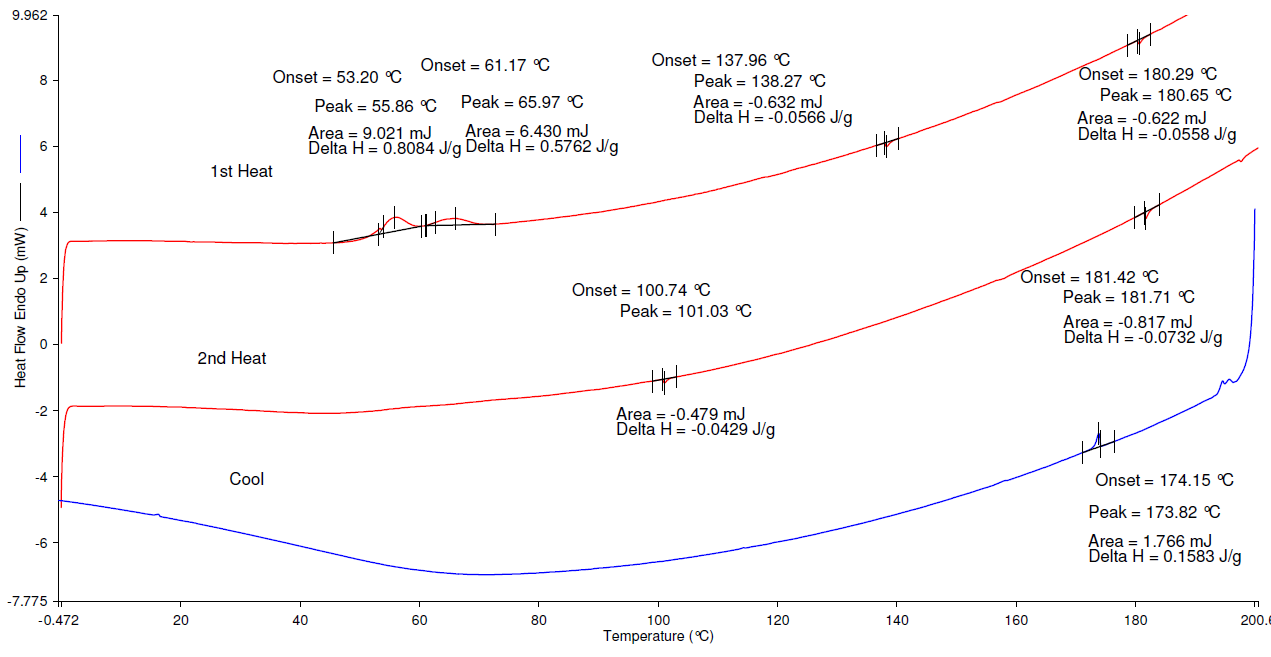


**Figure S7**: Differential Scanning Calorimetry heat flow vs. Temperature cooling curve for polymer (**2**) (poly(sulfur-*co*-bisphenol A dimethacrylate) with heating curves (red) and cooling curve (blue).


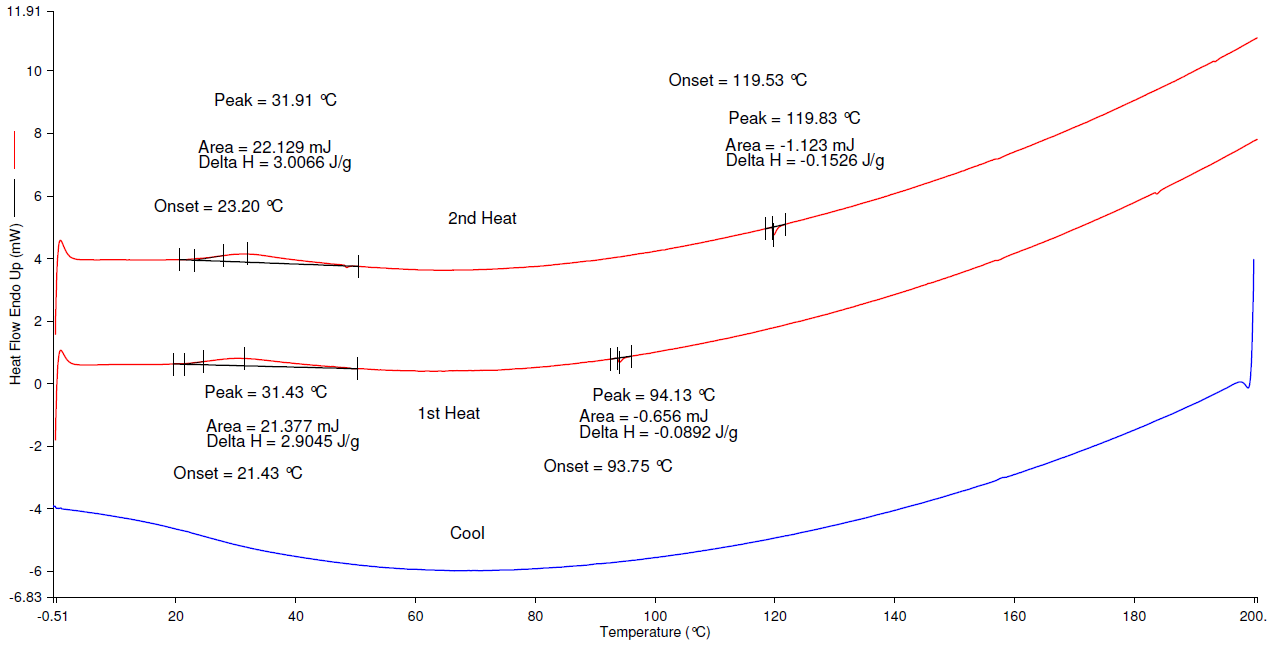


**Figure S8**: Differential Scanning Calorimetry heat flow vs. temperature cooling curve for polymer (**2**) ((poly(sulfur-*co*-bisphenol A dimethacrylate-*co*-1,3-diisopropenylbenzene), higher ratio of 1,3-diisopropenylbenzene with heating curves (red) and cooling curve (blue).


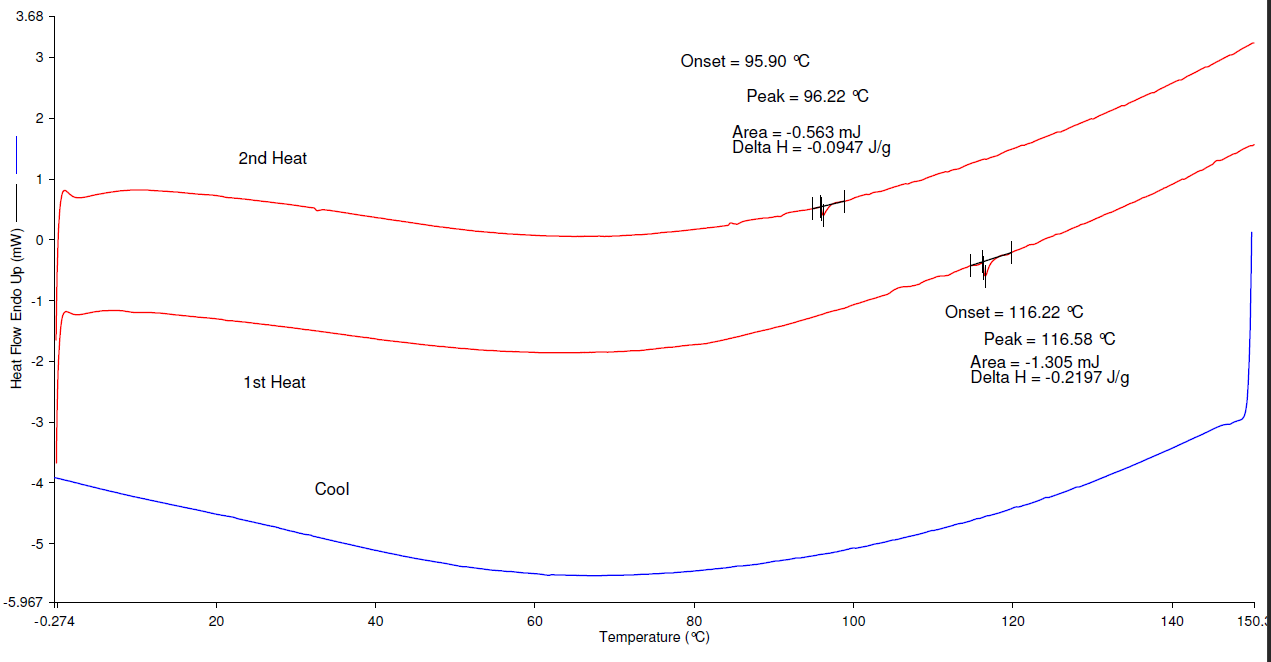


**Figure S9**: Differential Scanning Calorimetry heat flow vs. temperature cooling curve for polymer (**2**) ((poly(sulfur-*co*-bisphenol A dimethacrylate-*co*-1,3-diisopropenylbenzene), lower ratio of 1,3-diisopropenylbenzene with heating curves (red) and cooling curve (blue).


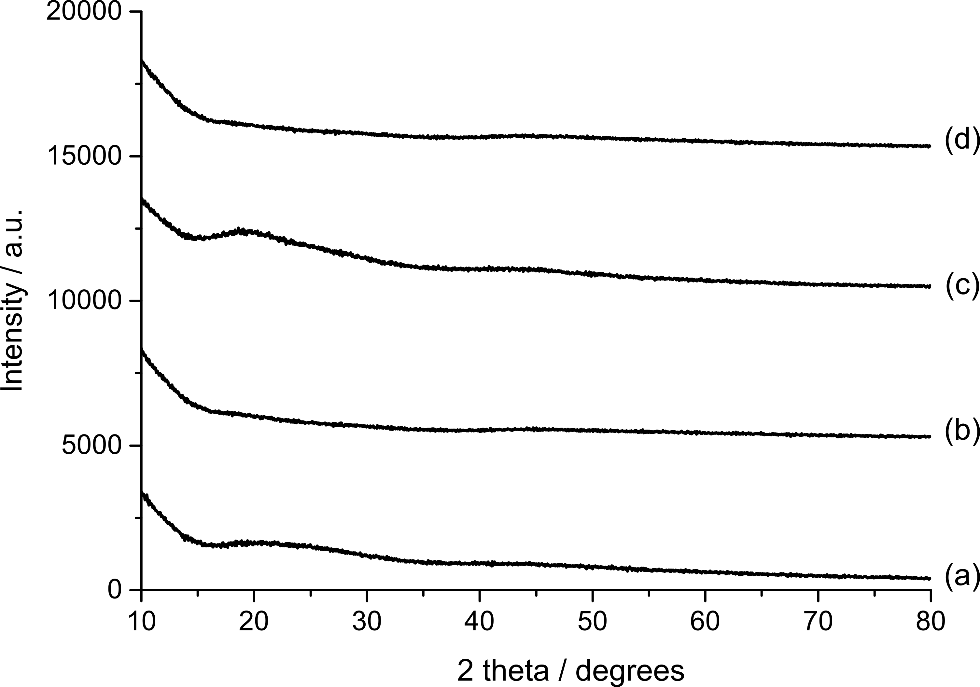


**Figure S10**: Powder X-ray diffraction patterns of: (a): poly(sulfur-*co*-1,3-diisopropenylbenzene), 50 wt. % ratio of linker (1), (b) poly(sulfur-*co*-bisphenol A dimethacrylate) (2), (c) poly(sulfur-*co*-bisphenol A dimethacrylate-*co*-1,3-diisopropenylbenzene) (4) and poly(sulfur-*co*-bisphenol A dimethacrylate-*co*-1,3-diisopropenylbenzene) (3).


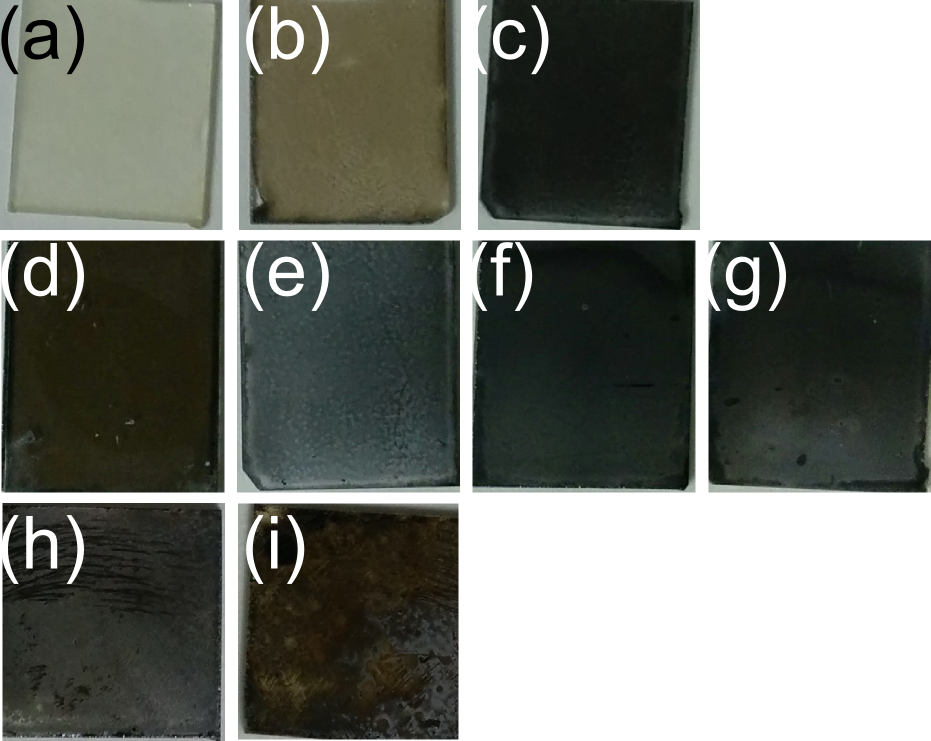


**Figure S11**: Photographs of prepared films. (a), (b) and (c) are the variable temperature series with polymer (**3**) (poly(sulfur-*co*-bisphenol A dimethacrylate-*co*-1,3-diisopropenylbenzene), higher ratio of 1,3-diisopropenylbenzene) at 50 °C, 100 °C and 150 °C respectively. d) 0.05 mmol, (e) 0.1 mmol, (f) 0.15 mmol and (g) 0.2 mmol of [Pb(S_2_COOct)_2_] are the films in the variable concentration study. h) is polymer (**3**) with [Pb(S_2_COOct)_2_] heated at 70° C for 3 days under nitrogen and (i) polymer (**1**) (poly(sulfur-*co*-1,3-diisopropenylbenzene) heated at 70° C for 3 days under nitrogen, forming a soft film.


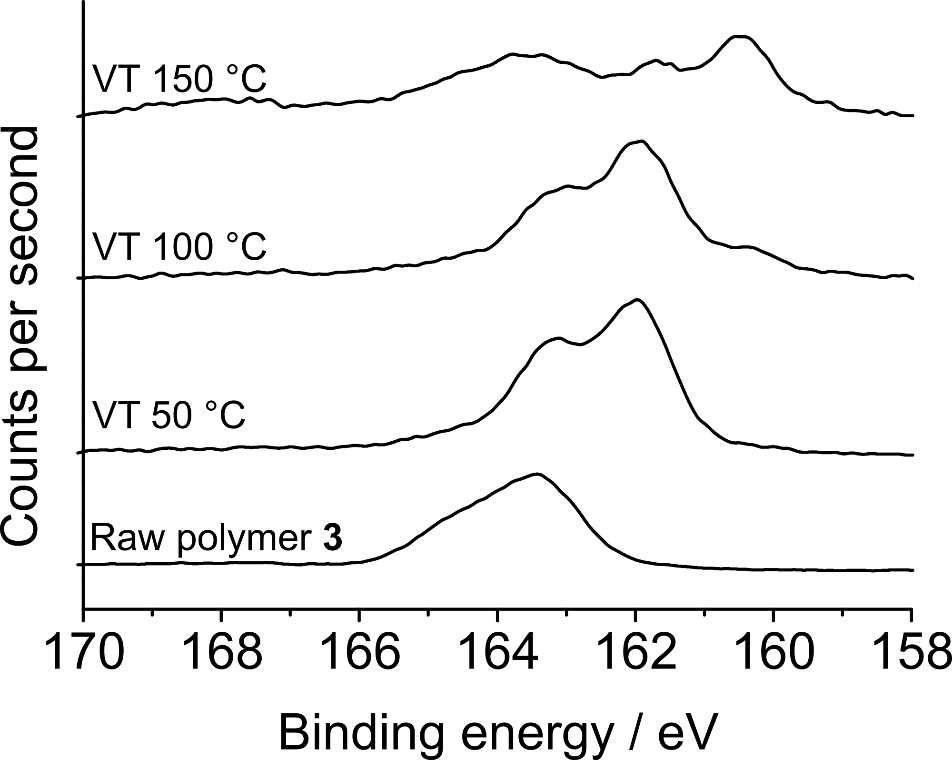


**Figure S12**: S2p regions showing the behavioural changes of [Pb(S_2_COOct)_2_] in poly(sulfur-*co*-bisphenol A dimethacrylate-*co*-1,3-diisopropenylbenzene), higher ratio of 1,3-diisopropenylbenzene, (**3**) with increasing temperature, showing the decomposition of the precursor and formation of PbS (160.2 eV).


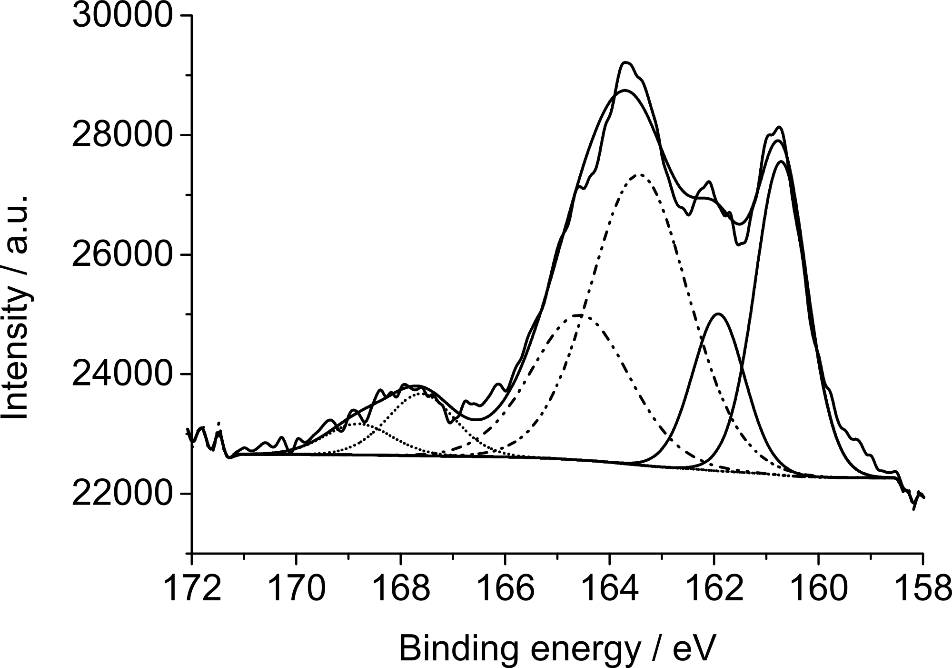


**Figure S13**: Fitted S2p regions showing S environments of [Pb(S_2_COOct)_2_] in poly(sulfur-*co*-bisphenol A dimethacrylate-*co*-1,3-diisopropenylbenzene), higher ratio of 1,3-diisopropenylbenzene, (**3**). The fitted regions (quoted values are position of S2p_3/2_) are attributed to PbS (160.8 eV), elemental sulfur (S_8_, 163.5 eV), and SO_x_ (167.6 eV).


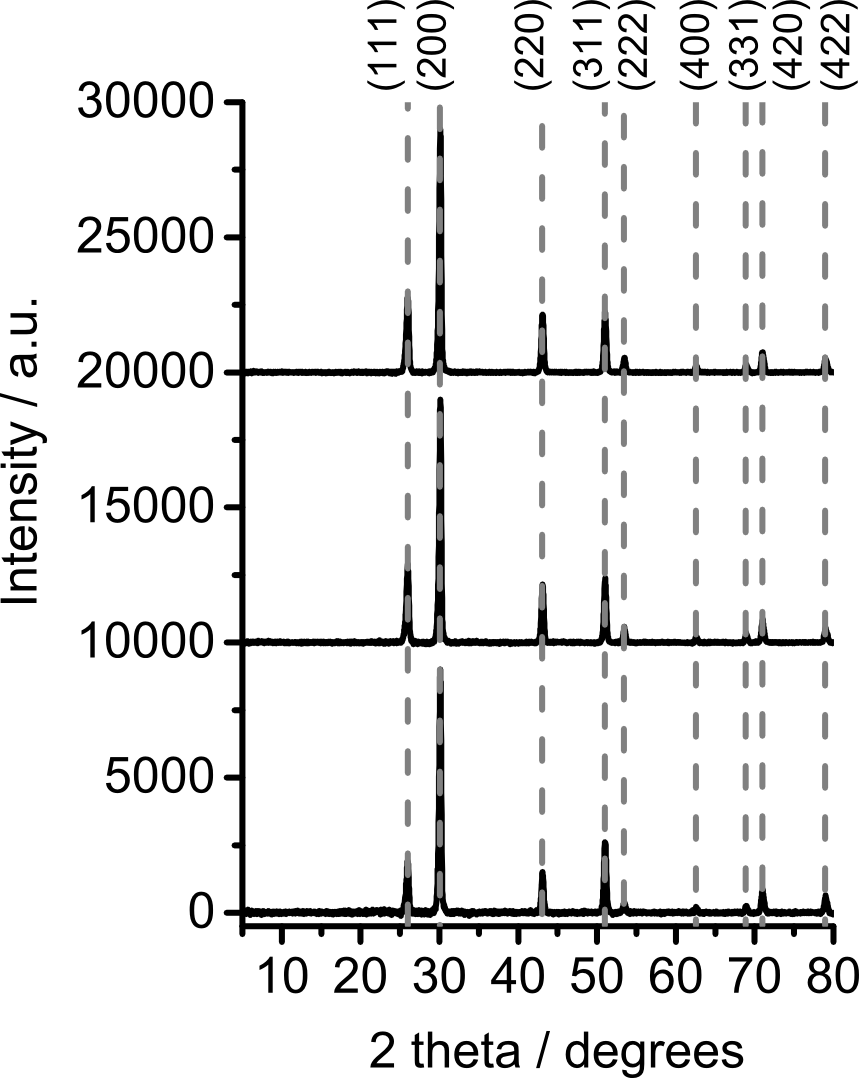


**Figure S14**: XRD patterns of the variable concentration series, (from bottom): 0.1 mmol, 0.15 mmol and 0.2 mmol. A reference pattern for PbS (galena) is superimposed (ICSD: 00-005-0592).


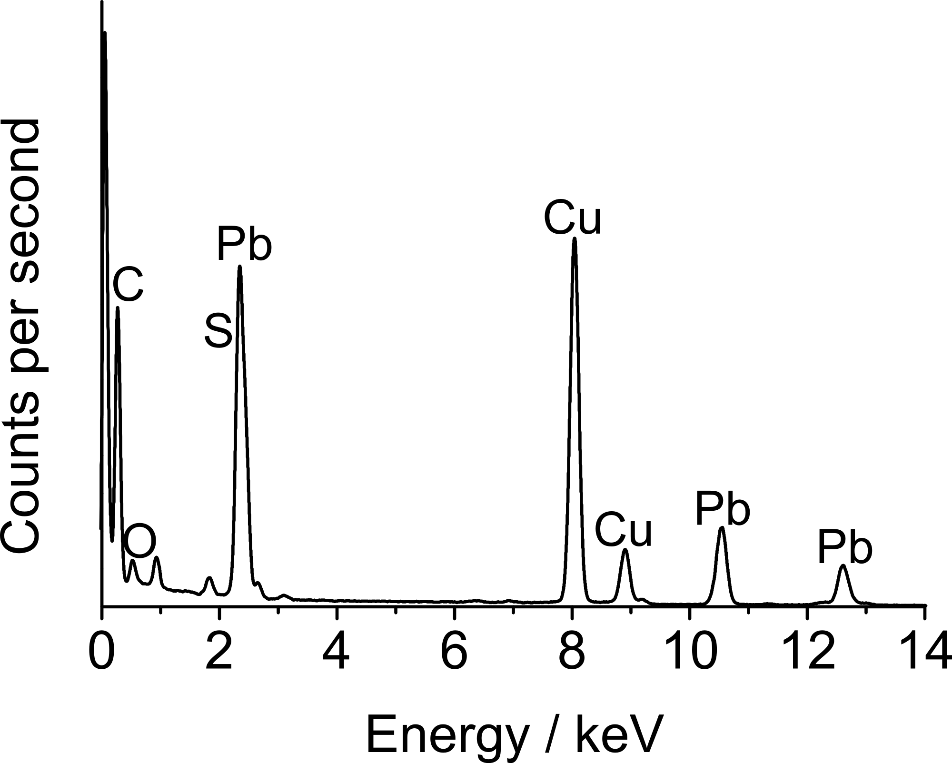


**Figure S15**: Exemplar EDS spectrum of 0.2 mmol of [Pb(S_2_COOct)_2_] in poly(sulfur-*co*-bisphenol A dimethacrylate-*co*-1,3-diisopropenylbenzene), higher ratio of 1,3-diisopropenylbenzene, (**3**) showing the presence of lead, masking the presence of sulfur.


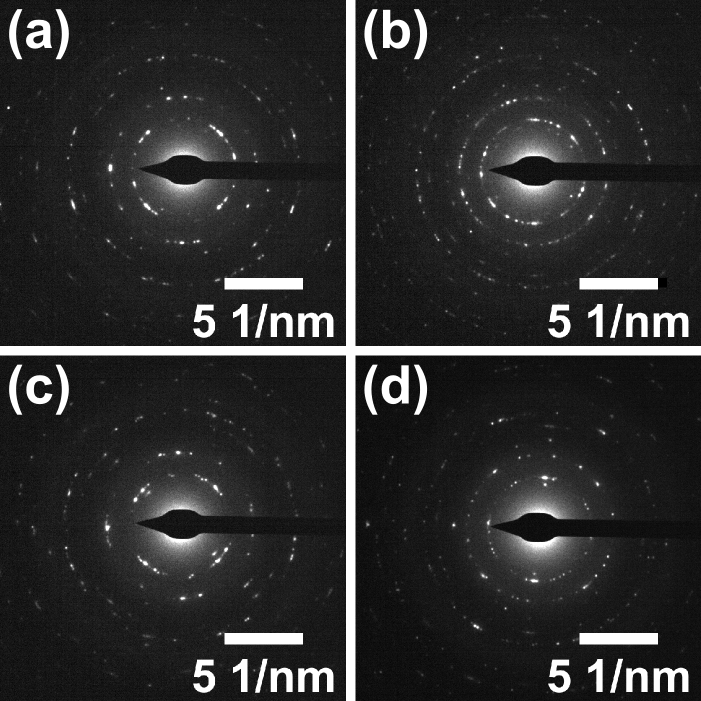


**Figure S16:** Selected area electron diffraction (SAED) patterns of PbS nanocrystals in the variable concentration range. Where a) 0.05 mmol, b) 0.1 mmol, c) 0.15 mmol and d) 0.2 mmol of [Pb(S_2_COOct)_2_] is used. All reflections were characteristic of PbS (galena).


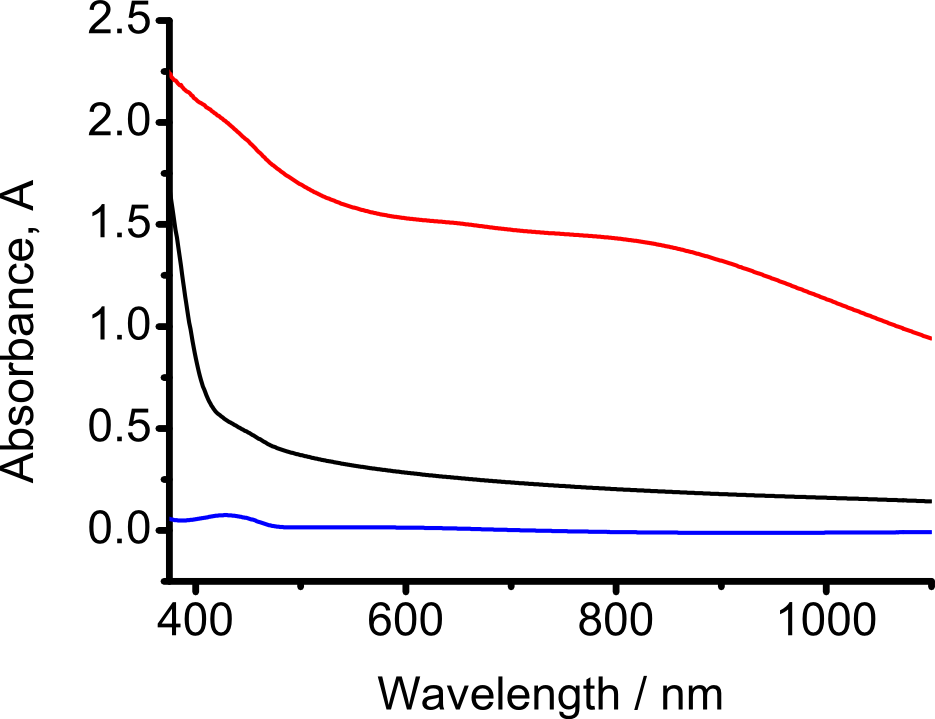


**Figure S17**: UV/vis spectra of: polymer (**3**) ((poly(sulfur-*co*-bisphenol A dimethacrylate-*co*-1,3-diisopropenylbenzene), higher ratio of 1,3-diisopropenylbenzene)) on a glass substrate (Blue), polymer (**3**) with [Pb(S_2_COOct)_2_], (Black) showing increased absorption and polymer (**3**) with full decomposition of [Pb(S_2_COOct)_2_] at 150 °C for 15 minutes under nitrogen (Red).

**Table S1:** A table equating sample number with composition and ratios of the different cross linkers used. It is noteworthy that the authors took the molecular mass of sulfur as 256.48 g mol^-1^ rather than 32.07 g mol^-1^.

| Sample Number | Sulfur, S_8_ / mmol | 1,3-diisopropenylbenzene / mmol | Bisphenol A dimethacrylate / mmol |
| --- | --- | --- | --- |
| 1 | 7.81 | 12.6 | 0 |
| 2 | 7.81 | 0 | 12.6 |
| 3 | 15.6 | 17.5 | 6.17 |
| 4 | 15.6 | 6.17 | 17.5 |
